# The Functional 3D Organization of Unicellular Genomes

**DOI:** 10.1038/s41598-019-48798-7

**Published:** 2019-09-04

**Authors:** Shay Ben-Elazar, Benny Chor, Zohar Yakhini

**Affiliations:** 1 School of Computer Science, Tel-Aviv University, Tel-Aviv, 6997801 Israel; 2 Department of Computer Science, Interdisciplinary Center, Herzliya, 4610101 Israel; 3 Department of Computer Science, Technion – Israel Institute of Technology, Haifa, 3200003 Israel

**Keywords:** Nuclear organization, Functional clustering, Statistical methods

## Abstract

Genome conformation capture techniques permit a systematic investigation into the functional spatial organization of genomes, including functional aspects like assessing the co-localization of sets of genomic elements. For example, the co-localization of genes targeted by a transcription factor (TF) within a transcription factory. We quantify spatial co-localization using a rigorous statistical model that measures the enrichment of a subset of elements in neighbourhoods inferred from Hi-C data. We also control for co-localization that can be attributed to genomic order. We systematically apply our open-sourced framework, *spatial-mHG*, to search for spatial co-localization phenomena in multiple unicellular Hi-C datasets with corresponding genomic annotations. Our biological findings shed new light on the functional spatial organization of genomes, including: In *C*. *crescentus*, DNA replication genes reside in two genomic clusters that are spatially co-localized. Furthermore, these clusters contain similar gene copies and lay in genomic vicinity to the *ori* and *ter* sequences. In *S*. *cerevisae*, Ty5 retrotransposon family element spatially co-localize at a spatially adjacent subset of telomeres. In *N*. *crassa*, both Proteasome lid subcomplex genes and protein refolding genes jointly spatially co-localize at a shared location. An implementation of our algorithms is available online.

## Introduction

Studying the co-localization of elements along the genome^1^ is used for providing evidence of evolutionary or mechanistic relationships between genomic elements and genomic organization. There are well established functional mechanisms that are known to interact in cis via genomic proximity, such as genes along an operon, promotors and their associated coding sequence, nucleosome modifications and proximal chromatin accessibility, etc. Studying trans interactions has remained elusive until recent technological breakthroughs that have enabled the assessment of the 3D structural properties of genomes. Chromosome conformation capture (3C) and methods derived therefrom (Hi-C)^2,3^ are, generally speaking, experimental protocols that yield a sparse map of paired sequencing read counts. These counts correlate with 3D spatial proximities between pairs of genomic loci^4^. These methods allow for a methodical examination of how the genome folds^5–7^ and how genomic elements co-localize to potentially interact in three-dimensional space^8–11^, opening the door to studying trans interaction systematically.

Hi-C has established a prominent and noteworthy contribution to our understanding of cis chromatin order and epigenetics with progress in the study and characterization of topologically associated domains (TADs)^12–14^. Such domains are typically presented as local triangle-shapes in a triangular view of the Hi-C interaction matrix, corresponding to local clusters of high intra-cluster, low inter-cluster read density. Studies pertaining to the underlying mechanism of TAD formation have implicated the contribution of CTCF and cohesin, key contributors to cell-type-specific genome conformation^15^. TADs are believed to form higher-order insulated intra-chromosomal neighbourhoods, regulating gene-enhancer interactions, and their disruption has been shown to cause disease^16^.

Imaging and Hi-C data, as well as data collected from related techniques, have been used to demonstrate co-localization of active genes in specific conditions and in a handful of organisms. The authors of^17^ were among the first to experimentally assess the nuclear localization of active genes. They applied FISH (fluorescence *in situ* hybridization) to provide evidence contrary to the hypothesis that active genes co-localize at the periphery of chromosome territories. A later study^18^, followed with a systematic analysis using independent 3C (chromosome conformation capture) and 3D-FISH experiments. Their results provided early evidence to the dynamic nature of co-localization of active genes. One purpose of this current work is to expand this investigation of co-localization in a more systematic manner. To achieve this, we developed streamlined algorithmic and statistical approaches as described herein.

**Figure 1:**
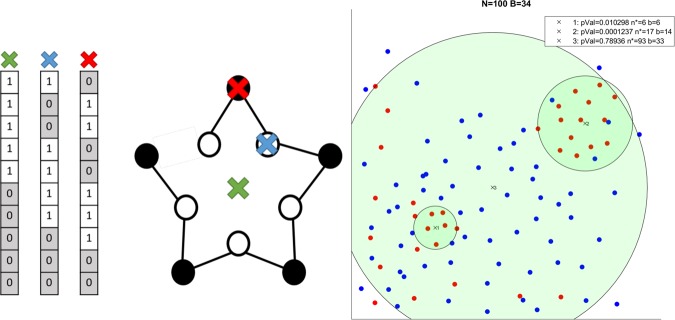
Left: A construct showing that (2D) spatial co-localization might not be identified by selecting positions along a 1D curve. Circles represent genomic bins. White circles contain TF targets; black circles are bins without TF targets. Red and blue ‘X’ represent both possible distinct pivots due to symmetry. On the left side we show the corresponding binary vectors reflecting the 2D (Euclidean) distance from each possible pivot. Green ‘X’ marks the optimal position (yielding the most significant mHG p-Value, see methods) and would not be identified with previous methods. Right: Showcasing three example pivots in a synthetic example. Three green discs representing three pivots (center of disc) with corresponding mHG p-values (in legend) and thresholds are reported. Red points are treated as binary ‘1’ in the corresponding *λ* vectors. x_3_ represents the center of mass of red points, illustrating its sensitivity to the distribution of red and blue points. x_1_, x_2_ show that the method can adjust to different densities in the data.

Transcription factories^19^ are an example of an established regulatory mechanism manifested as confined compartments within the nucleus, wherein transcription machinery recruits both cis or trans cofactors and genomic elements to regulate specific cellular functions^20–22^. Previous studies have attempted to address the task of statistically assessing the existence of transcription factories. The authors of^23^ compared the number of inter-chromosomal interactions in different functionally-related gene sets and observed statistical enrichment under the hypergeometric null model for interactions among transcription factor (TF) targets. However, a follow-up study^24^ argued that edges in the inter-chromosomal 3 C interaction graph are not statistically independent, as was assumed under the model used by^23^, and that co-localization events would therefore be over-counted. To correct for this issue, some studies^24^ applied a re-sampling procedure under which no signal for TF target co-localization was detected. Another study^25^ developed an extended approach that includes intra-chromosomal interactions along with a more elaborate sampling methodology which controls for local genomic structural features and applied this method to discover 3D co-localization of mutations in cancer and chromatin states. Studies from our group^26,27^ took a different approach to statistically assess transcription factories^23,24^ that avoids comparing between populations of pairwise proximities altogether, and so circumvents any statistical dependence issues that fail some earlier methods. Specifically, in the aforementioned work^26,27^ we compute our statistics independently on each genomic bin – a pivot point centered at some locus along the genome around which we measure the statistical significance of co-localization. Since this approach is only concerned with distances measured from a single fixed point, it avoids dependence issues related to working with all interaction pairs. For example, this approach never considers a triplet of significantly interacting genomic bin pairs (*i*, *j*), (*j*, *k*), (*i*, *k*) and therefore avoids dependence arising from transitivity, which was correctly pointed out by^24^. We rank all genes according to the number of interactions recorded between them and the pivot point under consideration. Using the ranked list of genes, we applied a statistical model to quantify whether targets from the functional set are significantly localized close to that pivot. We then apply additional safeguards to control for multiple hypotheses evaluated across different genomic bins and for events confounded by genomic proximity. The approach of^26,27^ is flexible in its inherent ability to detect partial co-localization of only a subset of the query set of TF targets, where approaches based on averaged Hi-C signal would require exponentially enumerating all possibilities. In addition to producing this subset, our method also produces the set of all genomic bins that geometrically reside within the convex subset of co-localized TF targets, but are not labelled as belonging to the query set. These bins could potentially hold elements that are functionally related to group in questions. A shortcoming of the above is that, in reality, co-localization needs not be geometrically restricted to a 3D point positioned precisely on a genomic locus but can be arbitrarily centred in space. Thus, events of significant colocalization may remain undetected by this method, as shown by the synthetic construction in (Fig. 1, Left). We later report a conceptually similar result on actual biological data for Caulobacter crescentus, further illustrating the need for a method that can overcome the shortcoming of such an approach. In both synthetic and real-data examples, none of the genomic bins yield a statistically significant co-localization result and such phenomena would be inadvertently ignored by methods that are limited to genomic bins as pivots.

In this work, we aim to extend our previous studies by removing the requirements for the pivot to reside on the genome. Our approach, as reported here, enables the study of co-localization of a set of genomic elements centred at arbitrary points in 3D space representations of Hi-C data. Investigating cis driven chromatin order, such as TADs, relies on the 1D topology of genomic order. Clearly, studying trans chromatin order, as in transcription factories, benefits from understanding the embedding of measured proximity data. We provide insights into the difficulty of solving this problem exactly and suggest several heuristics to approach it. We provide code and software implementing these approaches efficiently. In the discussion section, we compare our statistical enrichment approach to co-localization with a more simplistic sampling-based assessment. While a sampling-based approach will find some of the co-localization events, it will, as we show, miss several significant ones. Finally, we apply our method to multiple publicly available datasets across several species. Our analysis is able to uncover previously unreported cases of various genomic elements that appear significantly spatially co-localized. Co-localization alone cannot be used as direct evidence of an underlying mechanism due to potential confounding linkage. Although requiring additional experimental validation, these results shed new light on the genomic 3D organization of unicellular organisms.

## Materials and Methods

We present a statistical-algorithmic framework, referred to as *Spatial-mHG* (*smHG*, *in short)*, that can quantify patterns of spatial co-localization of binary-labelled elements.

Intuitively, our method scans an input set of 3D locations (for example, genomic bins in a 3D embedding of Hi-C data) labelled by some binary property, looking for ‘hotspots’. These are regions in which we observe an enrichment of ‘1’-labelled and a depletion of ‘0’-labelled genomic bins. Our method identifies hotspots as specified by 3D balls centered at pivot points. These events are statistically quantified for each pivot under a null model. We specifically use the, previously developed^28,29^, minimum hypergeometric null model. In the next two subsections we provide detailed formal definitions and analyse the computational complexity of providing exact solutions. We consider different algorithmic and heuristic strategies as well as statistical controls. This formal mathematical exposition can be skipped by readers who are not interested in such details of the methodology. The results section uses graphical representations that explain the nature of the results without relying on the mathematical details.

In the second part of this section, we list several Hi-C datasets as well as functional annotation sets explored in this study. We conclude this section by presenting a novel smoothed embedding approach that we applied for generating 3D configurations based on Hi-C data as input for *smHG*.

### Spatial-mHG: statistics

Consider a set of points in 3D with binary labels:

$${\mathscr{D}}={\{{x}_{i},{y}_{i}|\{{x}_{i}\in {{\mathbb{R}}}^{3}\},\,{y}_{i}\in \{0,1\}\}}_{1}^{N}$$

We define $B={{\rm{\Sigma }}}_{1}^{N}{y}_{i}$ to represent the number of ‘1’ labelled points in the data.

Let $p\in {{\mathbb{R}}}^{3}$ be some arbitrary point, also referred to as the ‘pivot’.

Define ${\lambda }_{p}=({y}_{{r}_{1}},\,{y}_{{r}_{2}},\,\ldots ,\,{y}_{{r}_{N}})$, the binary vector that satisfies:

$${\Vert p-{x}_{{r}_{1}}\Vert }_{2}\le {\Vert p-{x}_{{r}_{2}}\Vert }_{2}\le \ldots \le {\Vert p-{x}_{{r}_{N}}\Vert }_{2}.$$

That is *λp* is the binary vector induced by ranking points *x*_*i*_ according to their Euclidean distance from *p*. Further consider

$$\varphi (p)=mHG({\lambda }_{p})=\mathop{\min }\limits_{1\le n\le N}\mathop{\sum }\limits_{i={b}_{n}}^{{\rm{\min }}(n,B)}\tfrac{(\begin{array}{c}n\\ i\end{array})(\begin{array}{c}N-n\\ B-i\end{array})}{(\begin{array}{c}N\\ B\end{array})}$$

where $${b}_{n}={{\rm{\Sigma }}}_{{\rm{i}}}^{n}{\lambda }_{p}(i)$$.

*mHG* is a, previously published^26–29^, statistical framework that inspects prefixes of a binary vector, such as *λ*_*p*_, for overabundance of ‘eq. 1’ under a hypergeometric null model. Intuitively, the likelihood of an overabundance of ‘1’s is compared against a uniform distribution of such labels along *λ*_*p*_.

Since any two prefixes are statistically dependent, the resulting score requires a correction scheme to be applicable as a p-value. *mHG* corrects for multiple hypotheses by explicitly, and efficiently, computing the cumulative probability distribution function (CDF) for a given configuration of *N*, *B*. Querying the CDF at the resulting score yields a corrected p-value^29^.

In *smHG*, *ϕ*(*p*) would be small when ‘1’ labelled points co-localize around *p* (Fig. 1, Right).

Recall that we are interested in points that minimize *ϕ*(*p*), formally1

The *smHG* framework is therefore seeking pivots where a statistically significant *mHG* is obtained for the data, ${\mathscr{D}}$. As stated, solving (eq. 1) naively requires searching through all 3D space - a continuum of pivots. A relatively simple observation shows that the number of pivots that needs to be considered is actually finite. For every pair of points such that one is labelled as ‘1’ and the other as ‘0’ we can divide ${{\mathbb{R}}}^{3}$ using a plane that is perpendicular to their connecting line segment, and crosses in its middle. The arrangement of such (perpendicular bisecting) planes, or ‘bisectors’, tessellates the space into convex polygonal compartments, or ‘cells’. It is easy to see that given a single pivot from each cell (e.g. its centroid) we can cover all distinct binary vectors, *λ*_*p*_, for a given dataset. In Supplementary 10 we provide an exact polynomial bound on the number of pivots that produce distinct *λ*_*p*_ vectors as $${\rm{\Theta }}(\begin{array}{c}B(N-B)\\ 3\end{array})$$, leading to a worst case bound of *O*(*N*^6^), as previously described in^30^.

Unfortunately, from a practical perspective, this number of cells quickly becomes intractable even for moderately sized datasets, leading to statistical as well as algorithmic challenges. For a single cell (pivot) we can report precise *p*-values using the exact distribution of the mHG statistic^29^, however, there is a vast number of multiple hypotheses, namely cells, investigated in a single spatial-mHG instance as in (eq. 1). Characterizing a precise probability distribution for spatial-mHG remains a difficult task and so we apply FDR correction and report *q*-values. We also apply statistical assessment based on simulations as described below.

### Spatial-mHG: algorithmics and heuristics

An approach to evaluate spatial enrichment for a given set of labelled 3D data is a function ${\mathcal F} :{\mathscr{D}}\to [0,1].$ As indicated in the above discussion, the fast growth of the number of cells leads to algorithmic issues. Specifically, a naïve exhaustive approach for large *N*, although possible in principle, is practically infeasible due to the *O*(*N*^6^) complexity. In our analysis, we compare several heuristic approaches that aim to deal with this challenge. These approaches, denoted by *smHG*^*Grid*^ and *smHG*^*sample*^ correspondingly, provide an upper bound on *smHG*. As described, our methods are designed to detect significant results but cannot guarantee a recall of all significant results.

**Figure 2:**
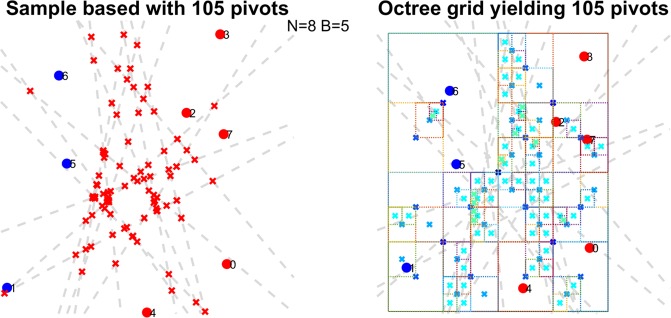
Illustration comparing implemented heuristics. Original points shown as red/teal and numbered from 0 to 7 where *B* = 4. 16 Bisectors are drawn as dashed gray lines, yielding 120 (closed) cells. Left (animation available as Supplementary Video 1): pivots generated in *smHG*^*sample*^ are red x’s. In this example our sampling algorithm is run to exhaustion Right (animation available as Supplementary Video 2): pivots generated in *smHG*^*Grid*^ are teal ‘x’s and corresponding dynamic grid structure colour coded by BFS depth in quad-tree. Here we stop the algorithm after yielding 120 pivots, illustrating the difference in behaviour to *smHG*^*sample*^.

See Supplementary 1,2 for discussion of the performance and trade-offs of the heuristics tested here and See Supplementary 3 for more technical notes on our experimental set up. An illustration summarizing the key differences between both approaches is available in Fig. 2.

#### Grid approach smHG^*Grid*^

We recursively iterate over a uniform 3D-grid. Namely, we partition space into eight disjoint, nested, cubes where the center of each cube is to be used as a pivot. This uses a common underlying data structure called octree^31^, and a branch-and-bound algorithmic approach. Let *C*_*t*+1_ be the t + 1^st^ - cube evaluated. *C*_0_ is the root node in the tree referring to a cube bounding our input data (with some slack to allow pivots outside the convex set to be considered). We dynamically build the octree while traversing it in a breadth-first manner by maintaining a priority queue. Let *OPT*(*t*) be the best observed *smHG* after *t* cubes are evaluated, and set $B{i}_{{C}_{t+1}}${bisectors that intersect with *C*_*t*+1_|bisectors that intersected *C*_*t*+1_’s parent cube}. Denote $smHG({P}_{{C}_{t+1}})$ the *smHG* score given by using the center of *C*_*t*+1_, ${P}_{{C}_{t+1}}$, as a pivot. We observe that at this point we have enough information available to compute a lower bound on the best theoretically-achievable *p*-value for all cells contained by the cube *C*_*t*+1_. If this lower bound is > *OPT*(*t*) we stop the recursion at *C*_*t*+1_ since no sub-cube can possibly improve on *OPT*(*t*).

Assume there exists a hypothetical pivot, ${p}^{hyp}\in {C}_{t+1}$, for which every bisector $bi\in B{i}_{{C}_{t+1}}$ is ‘satisfied’: Let $\{{x}_{1},1\},\{{x}_{2},0\}$ (W.L.O.G.) be the data points and labels which induced the bisector *bi*, ${p}^{hyp}$ ‘satisfies’ *bi* if $\Vert {p}^{hyp}-{x}_{1}{\Vert }_{2} < \Vert {p}^{hyp}-{x}_{2}{\Vert }_{2}$. Let *k* be the number of bisectors in $B{i}_{{C}_{t+1}}\,$that are not satisfied by ${P}_{{C}_{t+1}}$. We can compute $smHG({P}^{hyp})$ by exploiting the data structure used to compute $smHG({P}_{{C}_{t+1}})$. Intuitively, we append *k* ‘1’s after every valid prefix of ${\lambda }_{{P}_{{C}_{t+1}}}$ (such that *B* does not increase) and evaluate the resulting *mHG p*-value.

We note that this method guarantees a finite number of pivots, but each cell may be visited more than once. Details on this and more caveats are available in Supplementary 3.

#### Sampling approach smHG^*sample*^

Every three bisecting planes in general position (bisectors $${B}_{i}\triangleq {a}_{i}X+{b}_{i}Y+$$$${c}_{i}Z+{d}_{i}=0$$) intersect at a point, *p*_*B*_ = (*x*, *y*, *z*). We take an *∈*-step along the gradient of each of the three bisectors and average the resulting points to yield a pivot inside a cell *p*_*c*_. Formally,

$${p}_{c}=(\frac{1}{3}\mathop{\sum }\limits_{i=1}^{3}\frac{-({b}_{i}\ast (y+{\epsilon })+{c}_{i}\ast z+{d}_{i})}{{a}_{i}},y+{\epsilon },z)$$

This procedure defines a one-to-one mapping for every bisector-point-intersection to cells such that every such pivot point is “bottom-most” (w.r.t. dimension *y*) of some cell, as illustrated in Supplementary Fig. 8. With this in mind, we iterate over bisectors to yield combinations of three distinct bisectors and by doing so recover all “bottom-most” pivots exactly once.

Given an actual data instance, ${\mathscr{D}}$, we are interested in benchmarking the enrichment evaluated by any of the above approaches against adequate controls. To do so, we apply the following controls:

#### ‘Bead’ pivot control, denoted *Bead Control*

Uses every original *x*_*i*_ (‘beads’ along genome) as a candidate pivot, and only those. This is used to compare results with our previously published method^26,27^.

#### Genomic order control, denoted 1*D Control*

Uses every original x_*i*_ as a candidate pivot, but ranks according to 1D genomic distance (i.e. for *x*_*i*_, *x*_*j*_, rank by (*i* − *j*)), rather than, 3D, Euclidean distance. We restrict this analysis per chromosome where applicable, as genomic inter-chromosomal distance is undefined. This analysis is used to filter out results driven entirely by genomic enrichment, rather than spatial enrichment, as these are not the focus of this paper and can be identified without the need of Hi-C data or *smHG*.

#### Simulations control, denoted *P*_*sim*_

Runs 100X shuffles on the label vector, *y*, running both *smHG*^*grid*^ and *smHG*^*sample*^. *P*_*sim*_ is then reported as the empirical *SDF* where the population is comprised of 100 × min {*smHG*^*Grid*^, *smHG*^*sample*^} values. This evaluation is used as an additional approach of computing an empirically determined corrected *p*-value, since, as previously mentioned, *smHG* conducts multiple hypothesis testing (many dependent cells are treated independently) without an exact correction scheme.

### Hi-C datasets and annotation sets

We investigated several unicellular genomes and functional annotation sets, as follows:

- Bacteria: *C*. *crescentus*. Le *et al*.^32^ investigate expression of genes in chromosome interacting domains and their organization under a plectonemic model.
- Bacteria: *B*. *subtilis*. Marbouty *et al*.^33^ focus on the 3D architecture of the origin domain and its dynamics during the cell cycle.
- Yeast: *S*. *pombe*. Mizuguchi *et al*.^34^ experiment with Cohesin mutants illustrating its globule-formation function and discuss the role of heterochromatin in facilitating inter-chromosomal interactions.
- Yeast: *S*. *cerevisiae*. Duan *et al*.^35^ early work on structure reconstruction and the study of transcription factories.
- Fungi: *N*. *crassa*. Klocko *et al*.^36^ study sub-telomeric facultative heterochromatin and the impact of various histone modifications wildtype chromatin conformation.

Given an annotation dataset, namely one that induces binary labelling on genomic loci, we map annotation elements to genomic bins at the resolution, *N*, as provided in the aforementioned published Hi-C datasets. We filter out resulting annotation sets that map to less than four ‘1’ labelled bins (*B* < 4). We used several types of annotations, as applicable, for the different organisms.

#### Common annotation sets

- Gene Ontologies (GO) are acquired from^37,38^ for all five organisms.
- COGs/KOGs are acquired from^39,40^ for bacteria and yeast.
- Transcription factor target cohorts are acquired from^41^ for bacteria and from^42^ for yeast.

#### Differential annotation sets

We show how one can turn various types of genomic measurements into binary annotations that can be studied using our proposed framework. To illustrate this capability, we use the data published in *S*. *pombe*^34^ which includes the following datasets for both wild-type and mutants:

- CGH: Do copy number variations co-localize to some spatial locations?
- CGH data was binned to the same resolution as Hi-C, averaged by $\sqrt{\#mapped\,probes}$ in bin.
- Bins with less than 20 probes were removed. Resulting values, *V* = {*v*_*i*_} were binarized such that $${b}_{i}=\{\begin{array}{ll}1 & {v}_{i} > \mu +2\sigma \\ 0 & else\end{array}$$ where *μ*, *σ* are the mean and standard deviation of *V*, accordingly.
- Hi-C Data: Do genomic structural changes occur in spatial clusters?
- To evaluate differential Hi-C structures we compute Z scores from the Hi-C datasets of reference (REF) and variant (VAR). Then, per chromosome, we mask out (set as ‘0’) values in location *i*, *j* where *abs*(*i* − *j*) > 5 and compute the pairwise Euclidean distance between the masked vectors for locus *i* in REF and locus *i* in VAR and compute the Z scores on the results. Next, we binarize when $|Z| > 1.96$ to produce *y*_*i*_ for *smHG*. Intuitively, these are loci that have changed substantially in (local structure) curvature between REF and VAR. We use *x*_*i*_ from the embedding of REF.

### sNMDS smoothing of embedded Hi-C data

Embedding Hi-C data attempts to recover a 3D conformation, or ensemble of, that explains the observed data, with mounting qualitative evidence to support its reliability in capturing biological-structural phenomena^2,43–47^. We have previously^27^ demonstrated a quantitative advantage of using embedding distances over Hi-C read counts for the task of phasing haplotypes in a human genome, reinforcing its importance for denoising raw Hi-C read counts. We note that such embeddings cannot necessarily be conceived as representing an actual 3D genomic structure (see Discussion).

NMDS (Nonmetric Multidimensional Scaling)^48,49^ is a well-established embedding algorithm that iteratively minimizes a loss function measuring the *violations of ordinality* between the embedding and the input distances. Meaning, it attempts to find a conformation where the two closest points in the input will remain so in the embedding, and so forth. This property is desirable for *smHG* as it implies the embedding will directly optimize *λ*_*p*_ vectors for *p* ∈ {*x*_1_, …, *x*_*N*_}, to reflect the ordinality of observations as much as possible. Applying NMDS to Hi-C data often leads to unlikely discontinuities in the resulting configuration. Such discontinuities are especially evident in degenerate mapping of low-genomic-sequence-complexity regions and biased Hi-C measurements. For example, we may get consecutive genomic bins from the same chromosome that are unreasonably distant in space when compared to any other consecutive pair.

sNMDS (smoothed NMDS) iteratively corrects outliers in the embedding, enforcing smoothness for 1D genomic neighbours. Outliers are defined according to the distribution of distances between all genomically-consecutive bins (the discrete derivative) along the same chromosome. We compute Z-scores and provide thresholds as parameters that determine outliers (genomic discontinuities) for each iteration of the correction. These outliers are then corrected using linear interpolation. We demonstrate that this process results in qualitatively superior embedding configuration in Supplementary 5.

## Results

Using the method described herein we found evidence of functional 3D organization across multiple organisms and multiple functional annotation sets, illustrating the prevalence of structure-function relationship at a genomic scale, in unicellular organisms. Below we describe selected results chosen according to their statistical significance as well as according to their potential biological implications. We provide a supplementary table with more details for all results. as well as some descriptive meta-analysis is available in Supplementary 8. To further highlight the advantage of the grid method in identifying particular cases of spatial enrichment we performed an additional meta-analysis directly comparing the results among suggested heuristics in Supplementary 13. Finally, a discussion on several noteworthy negative results where functionally related elements did not appear to co-localize is available in Supplementary 9, for completeness.

### sNMDS results for Hi-C data of unicellular genomes

The first step of our approach is to apply sNMDS to Hi-C data and produce a 3D embedding configuration that is used to represent denoised distances from noisy measured population Hi-C read counts. We base our enrichment analysis on these configurations. These embeddings should not necessarily be considered as representing actual genomic 3D structure as further considered in the Discussion section. We apply sNMDS and *smHG* to elucidate distinct spatial enrichment patterns across multiple organisms and provide insights into the variability and prevalence of genomic functional organization across phyla. In the next subsections we list our key findings for each organism and discuss previously unreported phenomena detected as significant by *smHG*, as related to the functional 3D organization of the organisms studied.

### Caulobacter crescentus

**Figure 3:**
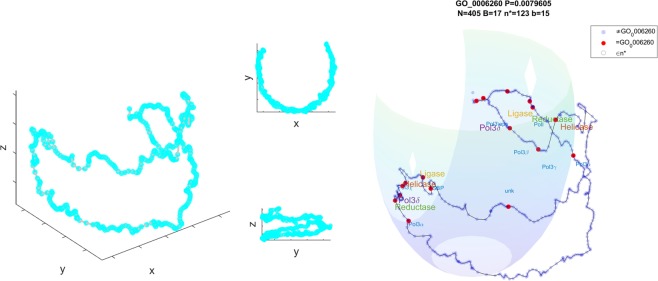
Left: sNMDS embedding of *C*. *crescentus* from three viewing angles. Right (animation: available as Supplementary Video 3): red spots are genomic bins which contain genes labelled as DNA replication genes under GO:0006260. The floating ‘x’ is the *smHG* optimal observed pivot. Translucent semi-sphere represents the ball induced by the *smHG* threshold. Gray circles indicate bins within the threshold and corresponding ball. Simplified gene labels in GO:0006260. Reductase in green, Helicase in red, Ligase in orange.

In Fig. 3 we present the sNMDS embedding of Hi-C measurements in *C*. *crescentus* (at synchronized cell cycle t = 0^32^), displaying a saddle-like, crescent structure, similar to its bacterial cell shape. A recently published^50^ high resolution structural study provided qualitatively similar models with experimental validation.

Genes annotated as elements of DNA replication (GO:0006260) appear polarized in two distinct sets along the replication axis (*smHG*^*Grid*^:[*P* < 6*e*^−6^;*Q* < 8*e*^−3^;*P*_*sim*_ < 0.01], *Bead Control*:[*P* < 0.02;*Q* < 0.32], 1*D Control*:[*P* < 0.03;*Q* < 0.85], Fig. 3, middle). Note that this is a real data example resembling the synthetic construction used in Fig. 1 in the sense that *smHG* finds an enrichment centered around a non-genomic pivot that is not evident under the bead pivot nor under the 1D genomic based approaches. Focusing on the individual gene families the observed dichotomy coincides with *ori* and *ter* locations, alluding to evolutionary pressure for duplicated machinery templates possibly related to the replication mechanism. A possible explanation of this observation can come from having a fall-back template for critical elements in the replication machinery in case of a stalled replisome blocking RNAP access^51^. We also observe more subunits from the DNA pol III family available near the Ori, which may relate to the fact that the cell exists longer in a state where these regions are replicated before meiosis.

The observed behavior of polarity along the replication axis appears to be a property of *C*. *crescentus*. We performed a meta-analysis of our results (Details in Supplementary 6) that illustrate that this property is consistent across available annotation sets and is significant (*P* = 0.01) under an appropriate statistical model.

### Bacillus subtilis

**Figure 4:**
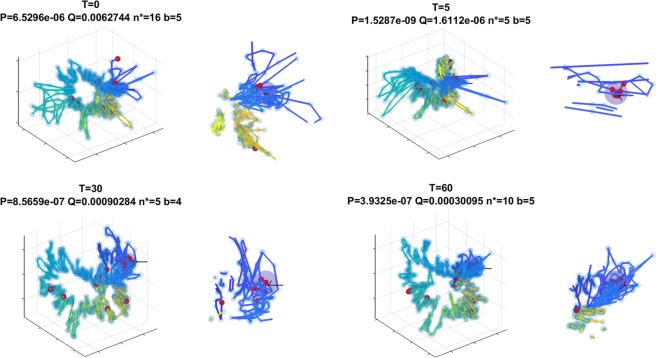
1 Left-to-right, Top-to-bottom (animation available as Supplementary Video 4): Embeddings of time-course Hi-C of *B*. *subtilis* at t = {0, 5, 30, 60} minutes after release from synchronized G1 into S-phase. Embeddings are aligned with Procrustes analysis. Color gradient along the chromosome is genomic position (showcases the circular nature of the chromosome). Red circles indicate genomic bins that contain gene(s) targeted by BSU00470 (Purine biosynthesis operon repressor). A single translucent ball in each subplot represents the *smHG* result (pivot and threshold mapped to radius). A black arrow points to the location of the ball. Figure depicts the dynamic nature of co-localization of the targets of the above TF. Next to each subplot we show a zoomed-in plot of the sites of detected co-localization.

In Fig. 4 we present four sNMDS embeddings of Hi-C data from available time-course Hi-C measurements in *B*. *subtilis*^33^.

Targets of transcription factor BSU00470 (Purine biosynthesis operon repressor) co-localization signal shifts and changes during cell cycle. We observe a substantial colocalization increase in *T* = 5 minutes after release from G1 into S-phase, as defined by the original report^33^. Results are summarized in Table 1 and visualized in Fig. 4, top right.

**Table 1:** *B*. *subtilis* BSU00470 (Purine biosynthesis) TF target co-localization dynamics during cell cycle.

T	smHG^Grid^	Bead Control	1D Control
0	*P* < 2*e*^−6^; *Q* < 0.04	*P* < 1*e*^−5^; *Q* < 0.007	*P* < 1*e*^−8^; *Q* < 1*e*^−5^
**5**	***P* < 1*e***^−8^**;** ***Q* < 1*e***^**−3**^**;** ***P***_***sim***_ **< 0.01**	***P*** ** < 1** ***e*** ^**−8**^ **;** ***Q*** ** < 1** ***e*** ^**−5**^	*P* < 1*e*^−8^; *Q* < 1*e*^−5^
30	*P* < 1*e*^−6^; *Q* < 0.02	*P* < 1*e*^−6^; *Q* < 1*e*^−3^	*P* < 1*e*^−8^; *Q* < 1*e*^−5^
60	*P* < 1*e*^−6^; *Q* < 0.02	*P* < 1*e*^−6^; *Q* < 1*e*^−3^	*P* < 1*e*^−8^; *Q* < 1*e*^−5^

Purine synthesis and salvage gene expression has been observed to fluctuate substantially during the cell cycle and is known to respond quickly to changes in pool availability^52–54^. We therefore observe a co-localization of purine biosynthesis targets in the cell cycle period when they are indeed observed as active. Gram positive bacteria, such as *B*. *subtilis*, have been demonstrated to have a strong strand-specific purine asymmetry, skewed positively to the leading strand and related to the mechanism of DNA replication^55^. The work by Nouri *et al*.^56^ showed that carbon metabolism in *B*. *subtilis* affects DNA replication rates. This may relate to our observation as purine biosynthesis requires the fusion of a pyrimidine ring with an imidazole ring and therefore has a higher carbon demand. We propose that there may exist a regulatory link between these phenomena, owing to the differences in strand replication progression that is mastered by the metabolism of purine and pyrimidines. The observed co-localization signal is facilitated via 1D as targets share an operon that appears to be spatially invaded by confounding genomic elements when *T* ≠ 5. Our analysis of the temporal dynamics of several TFs (further details in Supplementary 7) provides compelling evidence for the transcription factory model where genes can dynamically co-localize in or out of sites of transcription^57^.

#### Schizosaccharomyces pombe

**Figure 5:**
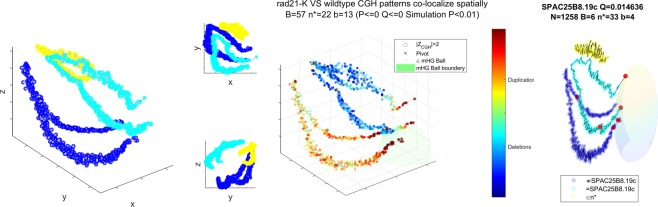
Left: sNMDS embedding for *S*. *pombe* with colour coded chromosomes. Middle (animation available as Supplementary Video 5): Bins are colour coded by average aCGH value, with marked outliers (opaque red for Z > 2 and blue for Z < −2). We can observe a weak duplication signal on ChrII, and deletion on ChrI, ChrIII. Strongest duplication is evident at the telomeres. Right (animation available as Supplementary Video 6): Red bins contain Loz1 transcription factor targets. The resulting *smHG* pivot and corresponding ball are visible containing 4/6 TF targets.

In Fig. 5 we present the sNMDS embedding of Hi-C measurements in *S*. *pombe*^34^, displaying a six-pronged claw shape. The authors of^58^ predicted a similar mitotic configuration in their proposed model.

Chromosomal rearrangement of rad21-K1 mutant (compared to Wild Type, based on aCGH data) are spatially co-localized near the telomeres (*smHG*^*Grid*^:[*P* < 1*e*^−300^;*P*_*sim*_ < 0.01], *Bead Control*:[*P* < 1*e*^−300^], 1*D Control*:[*P* < 1*e*^−8^;*Q* < 11*e*^−5^], Fig. 5, middle). rad21-K1 is a mutant selected for partial loss of function in a Cohesin subunit^59^. Cohesin is a protein complex implicated in being involved in the determination of chromatin architecture and mitotic domain organization^34,58,60,61^. Active chromosomal rearrangement near telomeres have been previously reported using Cohesin mutants in mice and molecular evolution studies in primates^62,63^. In a related observation we see that the transcription factor Loz1 has its targets spatially confined near the telomeres (*smHG*^*Grid*^:[*P* < 1.4*e*^−5^;*Q* < 0.02;*P*_*sim*_ < 0.02], *smHG*^*Pivot*^:*P* < 1*e*^−3^;*Q* < 0.1, *mHG*^1D^:[*P* < 1*e*^−2^;*Q* < 0.4], Fig. 5, Right). Two of its targets are SPBC1348.06c and SPAC977.05c, both known to be involved in telomeric duplication. Together, our results indicate a strong relation between a functional Cohesin complex and peri-telomeric integrity, which may be facilitated by DNA repair mechanisms operating during meiotic recombination.

**Figure 6:**
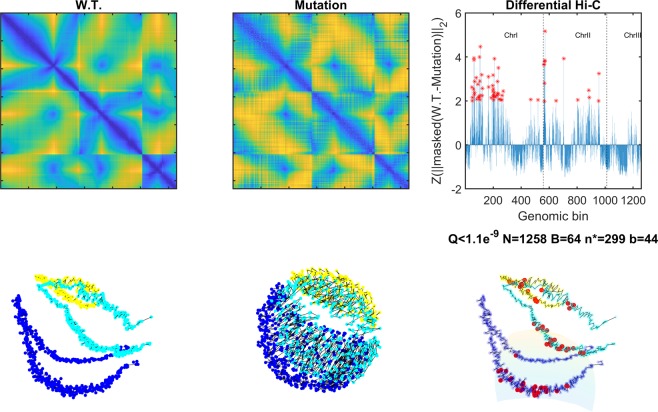
(Animation available as Supplementary Video 7) Left: Top – raw Hi-C read matrix for wildtype. Bottom – resulting sNMDS embedding. Middle: Top – Hi-C data for rad21-K1 mutant. Bottom – resulting sNMDS embedding. Right: Top – ΔZ-scores between both (masked) Hi-C datasets. Red asterix mark loci of Z > 1.96 change. Bottom – wildtype sNMDS embedding. Red bins indicate bins that substantially changed in their local structure according to our differential Hi-C analysis (detailed in Methods).

To further inspect the structural conformation changes in rad21-K1, we performed a differential Hi-C analysis (details provided in Methods). Our results show that the major changes in structure are localized and manifested primarily at the middle of each chromosome arm (*smHG*^*Grid*^:[*P* < 1*e*^−12^;*Q* < 1*e*^−7^;*P*_*sim*_ < 0.01], *Bead Control*:[*P* < 1*e*^−6^;*Q* < 1*e*^−3^], 1*D Control*:[*P* < 1*e*^−7^;*Q* < 1*e*^−5^], Fig. 6). The authors of^64^ present qualitatively similar interphase models.

#### Saccharomyces cerevisiae

**Figure 7:**
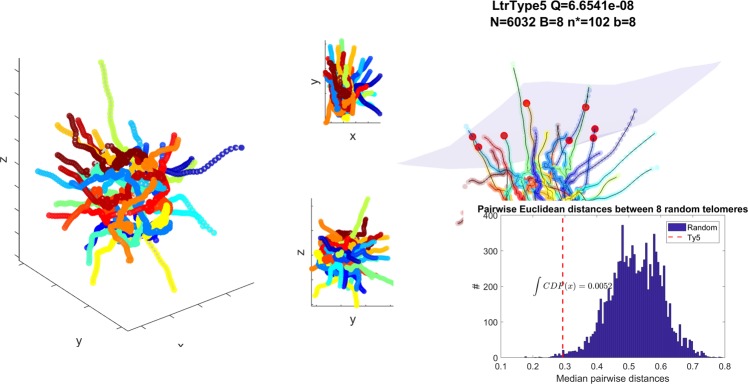
Left: sNMDS embedding for *S*. *cerevisiae* with 16 color-coded chromosomes Right (animation available as Supplementary Video 8): Opaque red colored bins contain Ty5 family LTRs. Inset shows the distribution of mean pairwise Euclidean distances for $$(\begin{array}{c}32\\ 8\end{array})$$ telomeres. Red dashed vertical line indicates mean pairwise Euclidean distances for the 8 Ty5 bins. An empirically determined cumulative distribution function evaluated at this point yields *p* < 0.007.

In Fig. 7 we present the sNMDS embedding of Hi-C measurements in *S*. *cerevisiae*^35^, displaying a Rabl^65^, Water-lily conformation. This result is qualitatively consistent with previously published models^26,61,66^.

*S*. *cerevisiae* long terminal repeats (LTRs) have been categorized to five distinct families, each with different properties^67,68^. We observe a previously known preference of family Ty5 to associate to peri-telomeric regions (*smHG*^*Sample*^:[*P* < 1*e*^−13^;*Q* < 1*e*^−7^;*P*_*sim*_ < 0.01], *Bead Control*:[*P* < 1*e*^−7^;*Q* < 1*e*^−3^], 1*D Control*:[*P* < 1*e*^−3^;*Q* < 0.04], Fig. 7). While this association was already known, we offer a refinement in such that the 8 annotated Ty5 LTR elements tend to co-localize at a specific hemisphere of the nucleus, on chromosomes III (3 instances), V (2 instances), VII, VIII and XI. We present the likelihood of such an event to be random in Fig. 7, Right inset. We shuffle (10,000 times) the assignment of Ty5 elements to different telomeres and compute the median of their pairwise Euclidean distances. The resulting empirical CDF at the unpermuted (observed) point yields *p* < 0.007. We propose that this co-localization phenomenon occurs due to the mechanism by which retrotransposons propagate. The probability of a transposing element to integrate in a potential target site is inversely proportional to the distance it needs to travel from its source.

### Neurospora crassa

**Figure 8:**
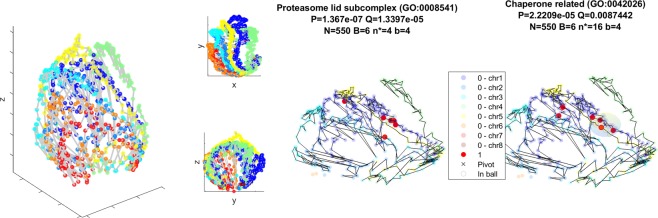
Left (animation available as Supplementary Video 9): sNMDS embedding of *N*. *crassa*. Middle & Right (animations available as Supplementary Video 11 and Supplementary Video 12): Only subset of bins containing mappable genes with GO terms are shown. Red coloured bins contain genes with GO (gene ontology) annotation GO:0008541 and GO:0042026, “Proteasome lid subcomplex” and “Protein refolding” (Chaperone related), accordingly. A black ‘x’ and translucent sphere depict the resulting *smHG* position and radius (recovered by mapping mHG threshold back to distance from ‘x’) for each figure.

In Fig. 8 we present the sNMDS embedding of Hi-C measurements in *N*. *crassa*^36^, displaying a balloon-like shape.

Protein folding genes and Proteasome lid subcomplex genes are poised to collaborate by genomic co-localization. In our analysis we observe both gene ontology terms (8541, 42026) to individually co-localize spatially (*smHG*^*Grid*^:[*P* < 1*e*^−9^;*Q* < 1*e*^−3^;*P*_*sim*_ < 0.01], *Bead Control*:[*P* < 1*e*^−6^;*Q* < 1*e*^−3^], 1*D Control*:[*P* < 1*e*^−6^;*Q* < 1*e*^−4^] and *smHG*^*Grid*^:[*P* < 1*e*^−5^;*Q* < 0.02;*P*_*sim*_ < 0.01], *Bead Control*:[*P* < 1*e*^−4^;*Q* < 1*e*^−2^], 1*D Control*:[*P* < 1*e*^−3^;*Q* < 0.02] accordingly, Fig. 8, Right). Upon inspecting the resulting pivot locations and the sizes of enrichment balls they appear similar to one another. To further validate this result, we compute *smHG on* the union of both GO term targets resulting in ${B}_{{\cup }^{}}=10$, indicating 2 bins overlap. We run *smHG* on the union without providing an exact statistical model to treat these overlaps, providing an upper bound on the *p*-value (*smHG*^*Grid*^:[*P* < 1*e*^−8^;*Q* < 1*e*^−4^;*P*_*sim*_ < 0.01], *Bead Control*:[*P* < 1*e*^−7^;*Q* < 1*e*^−4^], 1*D Control*:[*P* < 1*e*^−6^;*Q* < 1*e*^−4^]). Additionally, we fixed the 6 target bins of GO: 0042026 and randomly picked 6 targets, computing the mean pairwise distances between both sets of points. The tail of the empirical distribution yielded *CDF* < 1*e*^−300^ when evaluated at the pairwise distances between GO: 0042026 targets and GO: 0008541. These validations further illustrate that these are independent genomic sites with overlapping spatial co-localizations. In summary, we observe a significant co-localization of Proteasome genes as well as of Chaperone genes and furthermore, these two putative transcription factories are spatially close to each other. It has been previously observed that both machineries are intertwined, where chaperones mark for degradation by ubiquitination, physically deliver and interact directly or via coefficients with the proteasome machinery^69,70^. Our observation suggests that both mechanisms are tightly coupled on the genomic level thereby offering an increased linkage and co-regulation.

## Discussion

In this work we have developed and implemented methods for assessing the statistical significance of spatial co-localization in binary data specified for 3D co-ordinates which overcomes the limitation of being constrained to ‘Bead’ pivots. Our code is available to the community. We have applied our methods to analyse several Hi-C datasets from unicellular genomes and report statistically significant results detailed above.

Our analyses are performed on previously published “population Hi-C” datasets. That is, Hi-C read counts correspond to evidence of proximity events sampled from millions of independent genomes of distinct biological cells. In this work, as well as in some other Hi-C literature, results are based on analysing such population data. The underlying biology may therefore be obscured by the non-homogeneous character of the data. To mitigate the underlying variability, we focus on analysing datasets of monoclonal single-celled organisms at synchronized cell-cycle stages and under shared environmental conditions. We therefore expect reduced effects coming from genetic, functional and environmental non-homogeneities. Nonetheless, other factors that contribute to variability remain, and enrichment results should only be interpreted as statistical observations derived from 3D configurations based on sampled population measurements. Applying our methodology on more complex organisms, such as Humans, will require several adjustments: First, methods that sample homogeneous cell populations, or single-cell methods. Next, correctly embedding a polyploid genome. Third, adjustments to the statistical model of mHG to better reflect the availability of gene copies in a gene set. Finally, mitigating the complexity issues discussed above at larger genome scales by developing more advanced heuristics.

Furthermore, we base our analysis on 3D configurations derived from population data as above. sNMDS embeddings probably do not represent the genome structure of any individual biological cell or population member. The spatial manifold in which elements are embedded cannot necessarily be directly interpreted as physical 3D space. Instead, it serves as an abstract ‘latent’ space, primarily useful for mapping Hi-C data to the geometry required for our statistical 3D enrichment methods, while smoothing out the noisy character of Hi-C read counts. The approach here could be re-interpreted not as identifying “colocalization” of sets of genomic elements from a spatial model of a genome, but simply testing for statistical enrichment at the level of bulk contact frequency, which hints at some cases of colocalization. We view the fact that resulting embeddings visually correlate with our expectations of polymer behaviour without being strictly enforced in the embedding process along with the observed statistically significant *smHG* results as added qualitative evidence of a population-driven structural signal of genome organization. A quantitative quality control analysis of the embedding process, reinforcing the selection of embedding algorithm and parameters, is displayed in Supplementary 11.

The algorithmic approach we take here is heuristic since the exact calculation of the best *smHG* pivots in the data corrected for multiple testing is complex. It is clearly a low polynomial search problem as indicated by the combinatorics of the bisector tessellation (see Methods), but still, for thousands of points (as in small genomes), this becomes an unacceptably long calculation. One may consider the use of a Voronoi tessellation. The latter has a far lower computational complexity. However, points in the same Voronoi cell can induce dramatically different rankings on the ‘0’s and ‘1’s, as we illustrate in Supplementary 12. Furthermore – the added complexity of correctly computing a statistically valid result by many repeats to correct for multiple testing, requires even greater time efficiency. We do analyse performance properties of our proposed heuristics, illustrating pros and cons of each.

Further investigation into heuristics may yield improved runtime performance for spatial enrichment methodologies. *Data reduction* methods^71^ may prove useful for filtering or replacing objects of interest (such as input points or tessellation cells) by applying clustering and selecting representatives. A specific noteworthy data reduction approach is to replace objects by fitting them with a density function^72,73^. A multiscale density-based representation^74^ could provide an efficient means of sampling candidate pivots from areas of interest. *Discrete non-convex optimization methods*^75,76^ such as applying local descent^77^ on the mHG p-value of neighbouring cells, may offer a mechanism to traverse between cells towards local minima, thereby enabling faster candidate elimination.

A simplistic approach to statistically assessing co-localization for a given set of genomic loci, *S*, would be to compare the average Hi-C read counts within *S* to averages obtained over a big number of randomly drawn samples of genomic loci with the same size, $|S|$. In Supplementary Fig. 13 we show an analysis comparing this approach with *smHG* on *B*. *subtilis* Hi-C data for targets of TF BSU29740 (ccpA), a LacI family transcriptional regulator. Our results in this analysis demonstrate the advantage of using *smHG* compared to a sampling-based approach which would not report this significant co-localization event. In general, from an algorithmic perspective, applying the sampling approach in a systematic way to find within a moderately enriched functional set (such as a TF cohort) the subsets that are more significantly enriched, is intractable. Specifically, for a TF cohort *S*, this is equivalent to enumerating all ${2}^{|S|}$ subsets.

We applied our statistical methods to several organisms across phyla. To summarize our observations: When analysing data from TF cohorts we find some of them to be spatially enriched, with evidence that functionally related cohorts can share a common transcription factory. We observe changes in co-localization patterns along cell cycle using time course data, providing evidence for transcription factory dynamics. We further show co-localized retrotransposon telomeric preference, potentially shedding new light on its mechanism of propagation. We observe an axial partitioning of replication machinery genes reinforcing evidence of a deep connection between genome replication and genome organisation.

Overall, we provide distinct lines of evidence for the role of spatial organization in unicellular organisms, illustrating *smHG*’s applicability to studying both cis and trans functional-structural relationships in genomes. Finally, our results and interpretation can benefit from follow-up studies and need to be experimentally validated.

## Supplementary information

Supplementary materials

Animated figures

Supplementary detailed results

## Data Availability

Spatial-mHG code is open source and available in the Yakhini Group GitHub repository (https://github.com/YakhiniGroup/SpatialEnrichment) along with animated 3D configurations and figures.
