# Supplementary material for: The Functional 3D Organization of Unicellular Genomes
Source: Sci Rep. 2019 Sep 4;9:12734. doi: 10.1038/s41598-019-48798-7 (PMC6726614; doi:10.1038/s41598-019-48798-7)
Supplement: Supplementary file 1 — Supplementary materials [file 41598_2019_48798_MOESM1_ESM.docx]

**The Functional 3D Organization of Unicellular Genomes**

Shay Ben-Elazar^1,*^, Benny Chor^1^ and Zohar Yakhini^2,3^

^1^ School of Computer Science, Tel-Aviv University, Tel-Aviv, 6997801, Israel

^2^ Department of Computer Science, Interdisciplinary Center, Herzliya, 4610101, Israel
^3^ Department of Computer Science, Technion – Israel Institute of Technology, Haifa, 3200003, Israel

* To whom correspondence should be addressed.
Tel: +972-523400043; Fax: +972-99567329; Email: [shay.benel@gmail.com](mailto:zohar.yakhini@gmail.com)

**Supplementary Materials**

1. **Empirical comparison of** $\boldsymbol{smH}\boldsymbol{G}^{\boldsymbol{grid}}$ **and** $\boldsymbol{smH}\boldsymbol{G}^{\boldsymbol{sample}}$ **on synthetic data:** To allow some degree of control on the optimal enrichment in a synthetically generated instance we provide the following protocol. Pick $\left\{ x_{i} \right\}^{N},c$ from a multivariate uniform, $\mathcal{U}(0,1)$, and desired minimal enrichment *p*-value, $p$. Assume $x_{i}$ is ranked by Euclidean distance to $c$, i.e. ${\|x}_{i}-c\left. \right\|_{2}\leq{\|x}_{i+1}-c\left. \right\|_{2}$. Enumerate all entries in the HGT table (Supplementary Figure 3) that are $\leq p$. Weight each entry with the number of possible non-decreasing paths that cross it (used in mHG multiple hypothesis correction, see (12, 13) for details) and apply importance sampling to select an entry proportionally to its weight. The entry corresponds to the underlying $b, n^{*}$ parameters. Generate $\lambda_{c}$ by creating a shuffled prefix vector with $b$ ‘1’s and $n^{*}-b$ ‘0’s, and ap pending a shuffled suffix vector with $B-b$ and $N-n^{*}$ ‘1’s and ‘0’s accordingly.
   We emphasize that this process only guarantees an *upper bound* on the OPT $smHG$ in this generated instance.

We run the process described above 10 times each for $N\in\left\{ 20,40,60,80,100 \right\}$ with desired $p<1e^{-3}$, and bound the runtime duration at 2 minutes. During the evaluation we record the best observed $p$ after $iter$ pivot evaluations. The results, presented in Supplementary Figure 1 show an advantage for $smHG^{grid}$ over $smHG^{sample}$ in both convergence time and magnitude (most evident for larger instances) of detected enrichment.

**
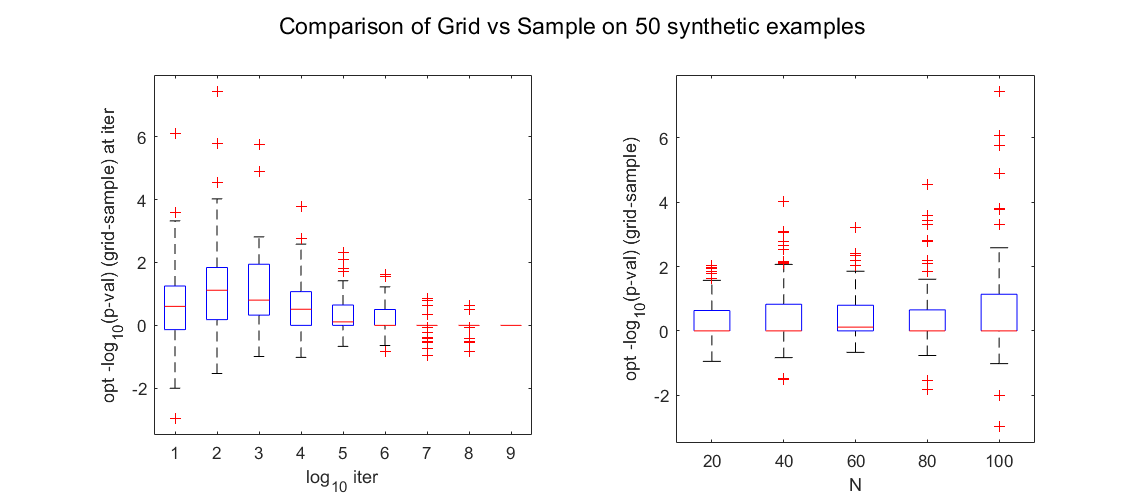
**

Supplementary Figure 1 Synthetic data comparison of $smHG^{grid}$ and $smHG^{sample}$ showing an advantage for $smHG^{grid}$ over in both convergence time and magnitude (most evident for larger instances) of detected enrichment.

1. **Additional notes on** $\boldsymbol{smH}\boldsymbol{G}^{\boldsymbol{sample}}$ **and** $\boldsymbol{smH}\boldsymbol{G}^{\boldsymbol{grid}}$ **differences:** During our work we have evaluated different approaches by simulating datasets with different tractable parameters where we could compare convergence times to optimal results. Evidently, while $smHG^{sample}$ provides a guarantee to exhaustively cover the exact number of cells, it appears to suffer from one major drawback by its hyper-sensitivity to the distribution of the data. In our simulations we sampled $x_{i}\mathcal{\propto U}(0,1)$, i.e. from a multivariate (2D or 3D) uniform distribution. Given $x_{i},x_{j}$ the midpoint of their connecting line segment (where the bisector lays) is an average of two uniform random variables, which, thus, in itself, is distributed under a special case of the (normalized) Irwin-Hall triangular distribution (n=2). This implies that uniformly generated data would have a substantially high concentration of bisectors at the center of the ambient space (0.5,0.5 for 2D and 0.5,0.5,0.5 in 3D). In turn, bisectors intersecting each other would yield a significant concentration of cells around that region. Since $smHG^{sample}$ picks cells uniformly, it would adopt this skew and over-represent this specific region of space. In a time-limited / truncated evaluation, we would miss evidence of co-localization in the periphery.
   $smHG^{grid}$ adopts a multi-resolution approach, forfeiting on theoretical benefits (that have little practical implications on large scale data) in order to inspect the input for possible co-localizations with increased granularity of over time.


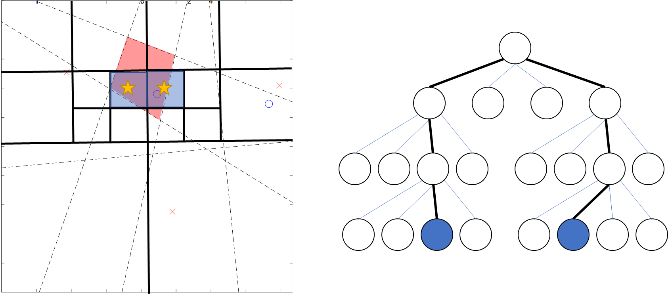


Supplementary Figure 2 showing how a single cell can be visited more than once by $smHG^{grid}$ as different branches of the Octree (2D shows quadtree) yield cubes that intersect it. (Left) 2D instance, bisectors shown as dashed lines. Cell of interest filled with red. Observe that there are bisector intersections that fall outside the axis limits and are not accounted for by this method (Right) corresponding tree graph of the resulting partitioning of running $smHG^{grid}$.

1. **Spatial-mHG technical notes:** We note that in our experiments we run the Grid and Sample heuristics for a bounded duration of 5 minutes each on a dedicated Azure StandardA8v2 machine.
   W.L.O.G. all input data is initially normalized to the unit ball and jittered to guarantee that the bisectors are in general position (with high probability). We also add a virtual sphere containing the normalized inputs, in order to make sure all cells are bounded.

A simplifying observation is that there are no intersections of more than three planes in the same point due to the following argument: Assume by contradiction that four planes intersect in a point. Choose one plane. Each of the three planes intersecting with it forms a line, and since they all intersect in a point. Since every line is a perpendicular bisector for points $x_{i}\in D$, the point of intersection is a circumcentre of a triangle where the triangle vertices are the bisected points in $D$. Since we only employ bisectors from pairs that are differently labelled (a ‘1’ and a ‘0’), this means that every pair of vertices in the triangle is differently labelled (or differently coloured). Since there is obviously no way to 2-colour a clique of size 3 (the triangle vertices), we contradict the original statement.

1. $\boldsymbol{smH}\boldsymbol{G}^{\boldsymbol{grid}}$ **recursion stopping criterion:** $\lambda_{p}$ can be illustrated as a non-decreasing path in an $B\times\left( N-B \right)$ matrix where each entry corresponds to a Hypergeometric CDF tail score and to a prefix of some possible binary vectors. For this entry, its Manhattan distance from the bottom left corner reflects the “number of draws” its row reflects the number of successes, and its column the number of failures (and implicitly the population size). The mHG score for a vector is the minimum value in the cells visited by its corresponding non-decreasing path. During an $smHG$ evaluation we track the optimal score observed, $p_{t}^{*}$. $p_{t}^{*}$ can be used to estimate the minimal number of cell traversals from $p$ that are necessary for obtaining a score that is better than $p_{t}^{*}$. This number is used as a stopping condition for the octree construction by comparing it to the number of bisectors crossing the cube for which our pivot is close to the ‘0’ coordinate than the ‘1’. If A visual representation is given in Supplementary Figure 3.

**
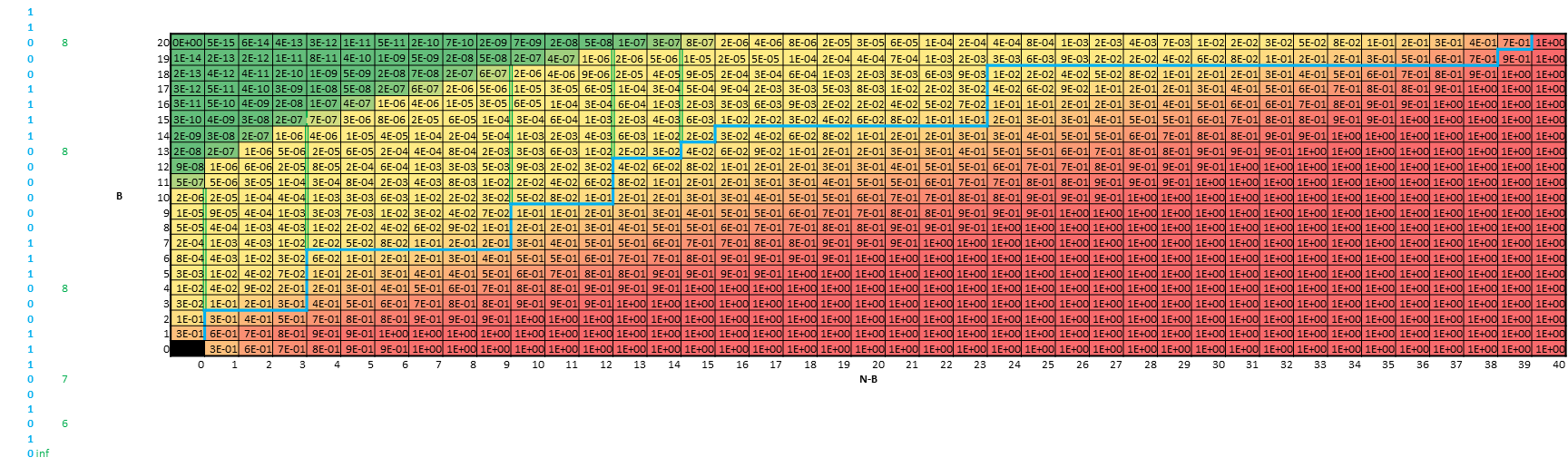
**

Supplementary Figure 3 An example HGT matrix depicting all possible binary vectors of size $N=60$ with $B=20$ ‘1’s. Every entry is colored proportionally to the hypergeometric CDF upper tail p-value. Blue path corresponds to some binary vector, $\lambda$, the prefix of which is displayed on the left. Vertical green lines emanating from this path towards the greed region of the table correspond to “minimal distance to $p_{t}^{*}=1e^{-6}$” at different thresholds in $\lambda$. The overall minimal distance in this case is $5.$

1. **sNMDS outlier correction scheme:** we present one of the NMDS resulting embeddings for B. subtilis time-course (at the 5-minute mark). We applied 2 iterations of smoothing with Z>4, Z>8, top row, visualized from left to right. Clusters identified in each iteration are and colour coded. We see that after each iteration the resulting genomic structure appears smoother and more coherent, unravelling more elaborate detail. We manually tune these hyperparameters to avoid having long stretches of the genome collapse to a line (example of a bad choice of parameters is presented in the bottom line).


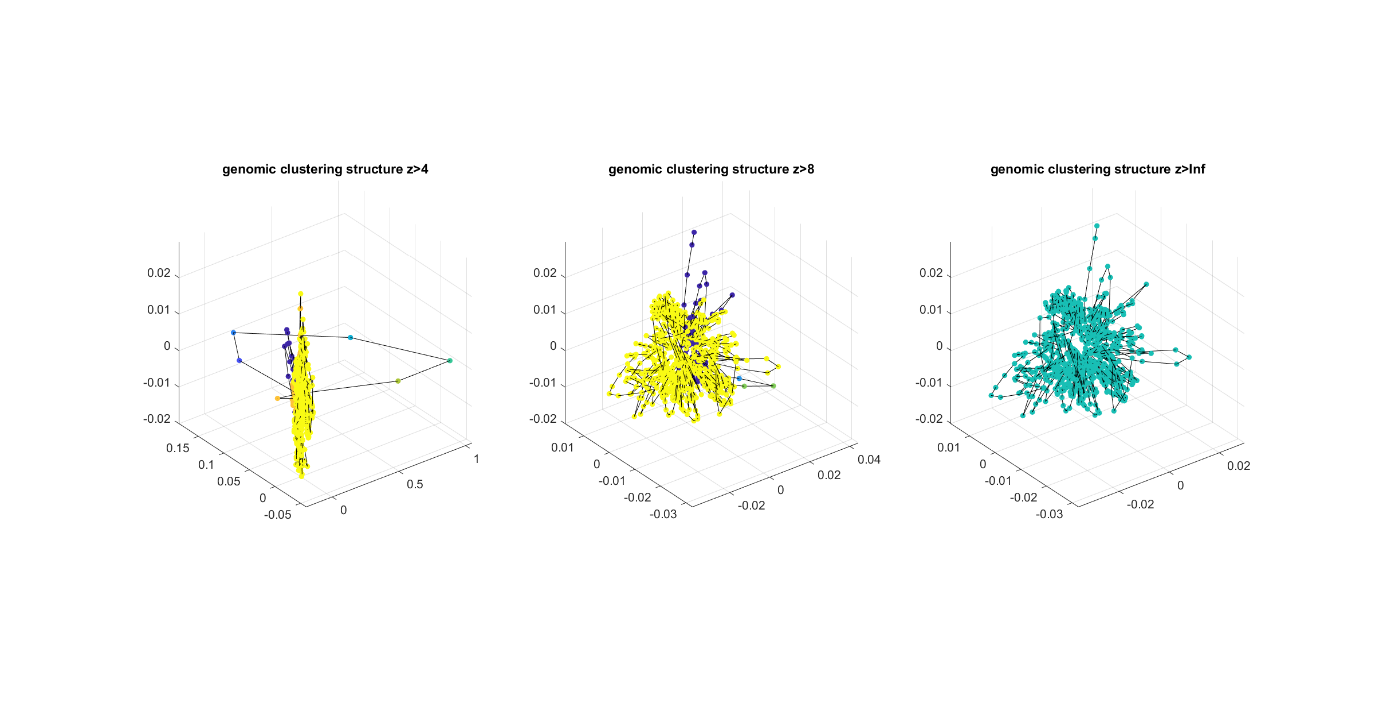


**
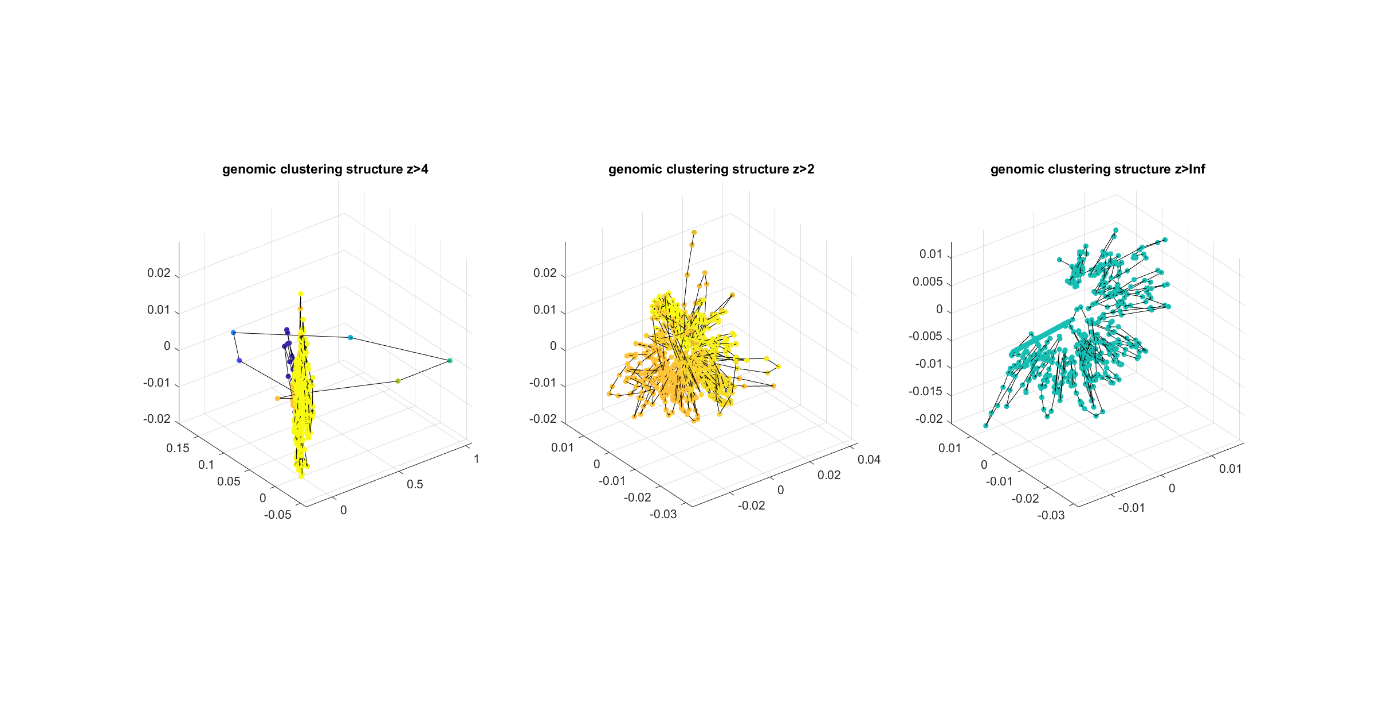
**

Supplementary Figure 4 (Top) showing two smoothing iterations of sNMDS on B. subtilis Hi-C data with Z>4 and Z>8. Genomic bins are color-coded by clustering them according to the Euclidean distances of consecutive bins. (Bottom) Same example with different parameters (Z>4, Z>2) showing the formation of an undesirable linear segment artifact. Note that this also impacts axis scaling as part of the manifold flattens.

1. **Principal directions of enrichment localization:** We weigh every resulting $smHG$ pivot across the investigated annotation sets with its corresponding $-\log q$-value. Next, we performed PCA on these weighted pivots, yielding the principal directions to explain the main variation in spatial enrichment in C. crescentus. The results, shown in Supplementary Supplementary Figure 5, left, illustrate a primary axis along the direction of the replication axis which explains 58% of the variance in enrichment directions. To quantify the significance of this observation we ran a simulation analysis, shuffling the *q*-value weights across pivots. The distribution of the resulting PC1 and PC2 explained variance are shown in Supplementary Supplementary Figure 5, right. We fit a multivariant gaussian and compute the density of the CDF at the empirically determined point (58%, 34%) showing that our observation is at the tail of the distribution, around 1% of simulated observations.


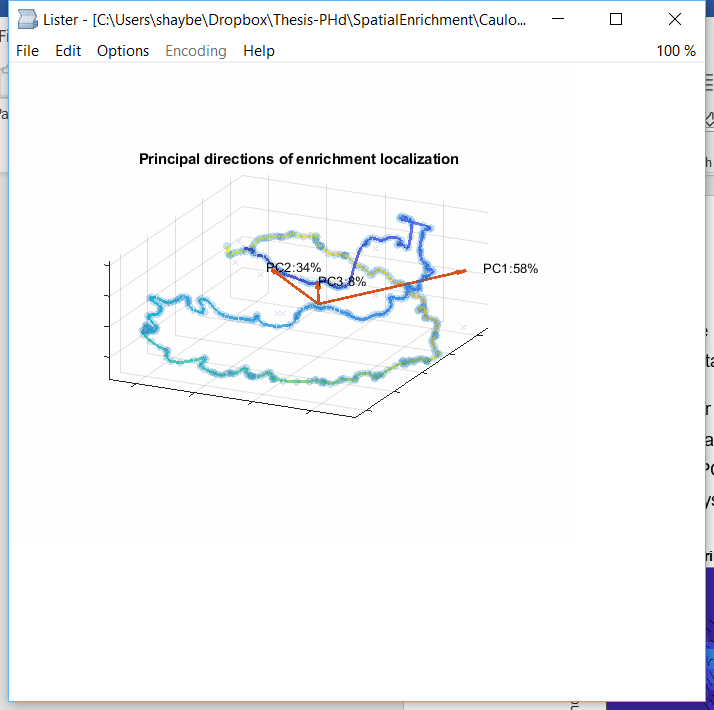
**

Supplementary Figure 5 (Left animation available as Supplementary Video 1s) Principal directions of enrichment as detected by our analysis. (Right) Permutation analysis showing there is a strong bias towards a single dominant axis of enrichment.

1. **Investigating temporal dynamics in time-course Hi-C data:** We plot each transcription factor’s $smHG$ -log *q*-value for each of the four available time-course Hi-C datasets. We map each enrichment to the set of genomic bins within the corresponding enrichment ball. Next for each temporally-consecutive pair of sets, we compute the Jaccard similarity to quantify the overlap between their targets. We then manually inspected TFs with temporal dynamics in both Jaccard and *q*-values.


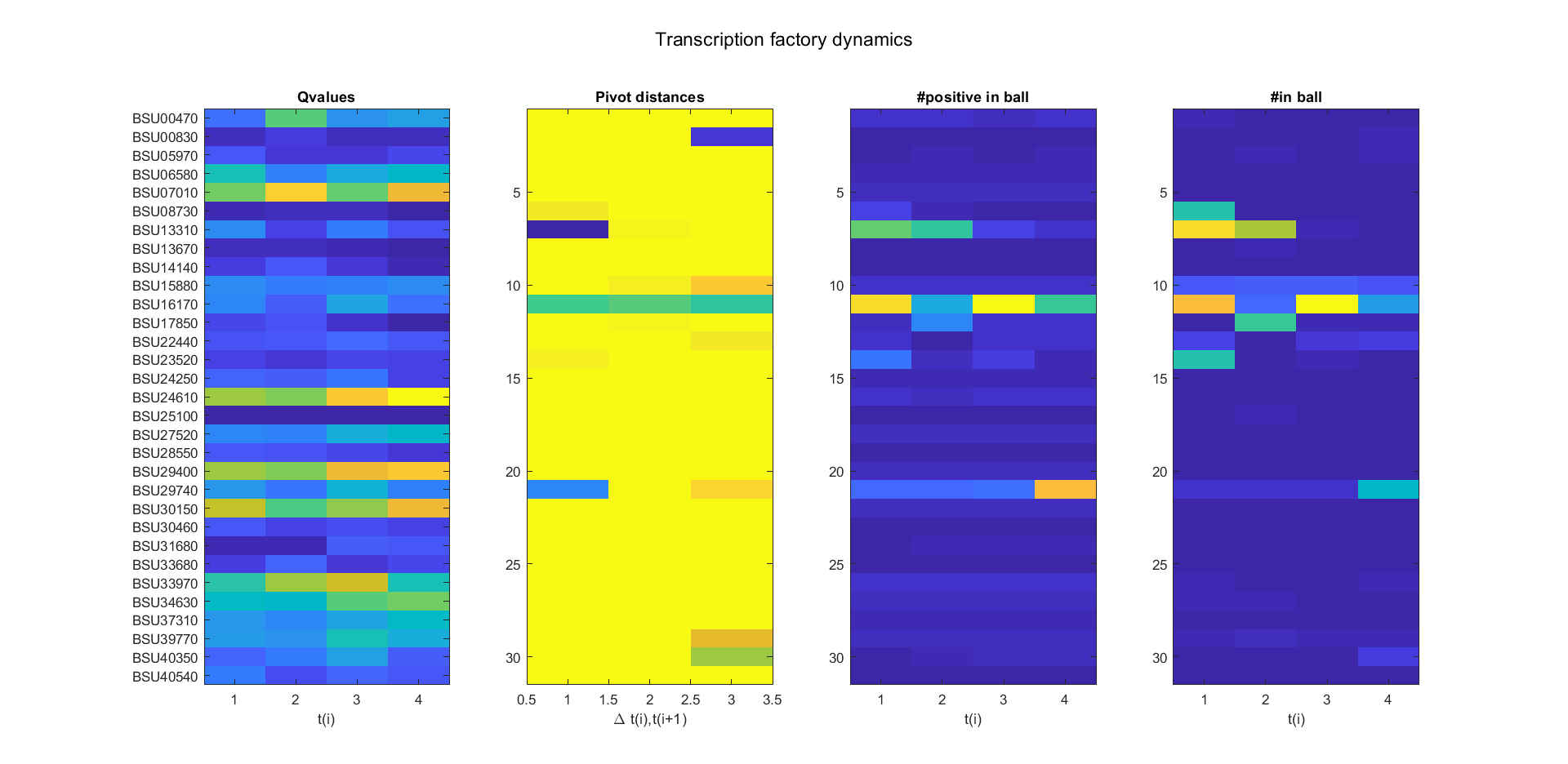


Supplementary Figure 6 Temporal dynamics in B. subtilis TF target smHG results. (Left) Enrichment Q values. (Middle-left) Overlap between bins inside the detected smHG enrichment ball for consecutive Hi-C datasets in the time course. (Middle-right) # of ‘1’s in the enrichment ball. (Right) number of genomic bins in the ball.

1. **Main results table:**Supplementary tables available in separate files. Supplementary Supplementary Figure 7 shows a meta-analysis of all *smHG* runs computed in this study.

**
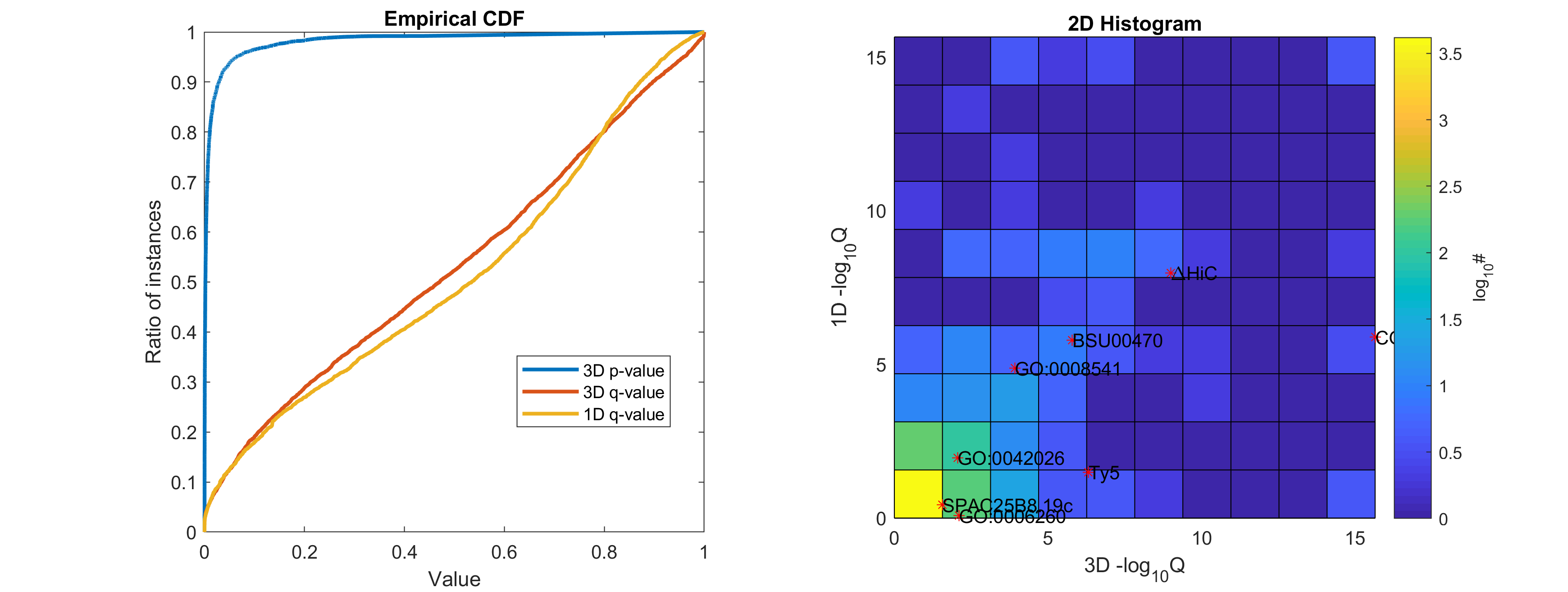
**

Supplementary Figure 7 Summary of 1D vs 3D Q values on all evaluated smHG instances. (Left) Empirical cumulative distribution plots of of 3D p and q values, and 1D q-value. We see FDR correction yields an empirical distribution that is approximately uniform, as needed, illustrating our sensitivity/specificity as a probabilistic model. (Right) a 2D histogram of 3D vs 1D q values. Results discussed in the main paper are overlaid and marked with a red asterix.

1. **Negative results:** in this section we detail a few noteworthy efforts that yielded no significant co-localization with the goal of illustrating diverse hypotheses that can be evaluated with our proposed framework.

Differential expression: We evaluated differential expression in two cases, and neither appeared to yield significant co-localization.

- The authors in (19) published a tiling array expression for Chr II in Pombe WT vs Rad21-K1. We average genes per bin, remove bins $\leq$90^th^ percentile in #mapped probes and with high variance within the bin. We compute the Z-score for differential expression on bins, and binarize with threshold $Z>1.96$. When limiting our analysis to this subset of bins we observe no spatial co-localization.
- The authors in (45) provide a Heat shock gene expression time course for S. cerevisae. We binarized the relative abundance values by averaging genes in bin and thresholding for $RA\geq5$, and we do not observe significant spatial co-localization under our model.

Other genomic element annotations:

- Pombe origins of replication (Ori) do not appear to spatially co-localization under our model.

1. **Exact bound on the number of cells induced by the intersection of planes:**

**Theorem I:** k lines partition the plane, $\mathbb{R}^{2}$, to at most $\left( \begin{aligned} k \\ 2 \end{aligned} \right)+\left( \begin{aligned} k \\ 1 \end{aligned} \right)+1$ distinct 2D cells.

**Corollary II:** $n$ points in $\mathbb{R}^{2}$ induce a partitioning of the plane to at most $\left( \begin{aligned} \left( \begin{aligned} n \\ 2 \end{aligned} \right) \\ 2 \end{aligned} \right)+\left( \begin{aligned} \left( \begin{aligned} n \\ 2 \end{aligned} \right) \\ 1 \end{aligned} \right)+1$ distinct 2D cells, when considering all PBHP (perpendicular bisecting lines, in the 2D case) between the points.

**Theorem III:** $k$ planes in $\mathbb{R}^{3}$ induce a partitioning to at most $\left( \begin{aligned} k \\ 3 \end{aligned} \right)+\left( \begin{aligned} k \\ 2 \end{aligned} \right)+\left( \begin{aligned} k \\ 1 \end{aligned} \right)+1$ 3D cells when considering the cells formed by the intersection of all PBHPs between pairs of points.

**Corollary IV:** $n$ points in $\mathbb{R}^{3}$, 3D Euclidean space, induce a partitioning to at most $\left( \begin{aligned} \left( \begin{aligned} n \\ 2 \end{aligned} \right) \\ 3 \end{aligned} \right)+\left( \begin{aligned} \left( \begin{aligned} n \\ 2 \end{aligned} \right) \\ 2 \end{aligned} \right)+\left( \begin{aligned} \left( \begin{aligned} n \\ 2 \end{aligned} \right) \\ 1 \end{aligned} \right)+1$ cells when considering the cells formed by the intersection of all PBHPs (perpendicular bisecting planes, in the 3D case) between the points.

*Theorem I Proof.* We denote points of intersection between two or more lines as ‘vertices’. Note that cells are equally defined by the lines that contain their edges (their boundary set), and by the vertices formed by the intersection of these lines. We define a one to one correspondence between cells and their bottom- most vertex (W.L.O.G. there is always such a vertex, otherwise we can tie-break arbitrarily, e.g. bottom-left vertex first). Assuming at most two lines intersect in any point, every point of intersection of lines serves as the lowest vertex of exactly one cell, thus there are $\left( \begin{aligned} k \\ 2 \end{aligned} \right)$ such cells. We now observe that some cells do not have a lowest vertex (they may be non-finite sets). To count them we “hallucinate” a k+1^th^ horizontal line below any intersection of our original k lines (see Supplementary Figure 8). To count the number of cells formed by the new line we assign each such region to the original line intersected to create it, arbitrarily on the vertex to its left. This process would end after k assignments with one region to spare. Thus, in total we have $\left( \begin{aligned} k \\ 2 \end{aligned} \right)+\left( \begin{aligned} k \\ 1 \end{aligned} \right)+1$ cells. Q.E.D. $∎$


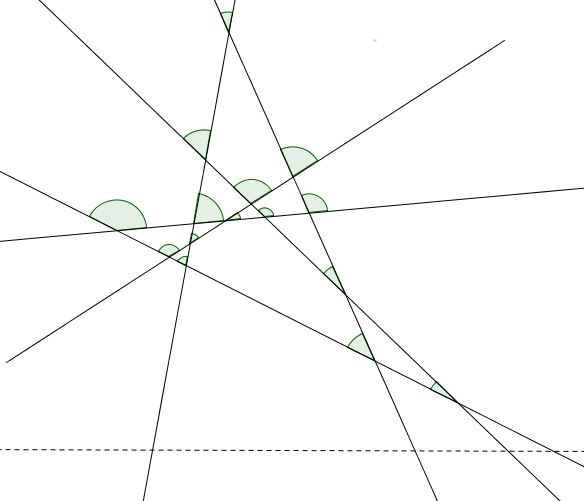

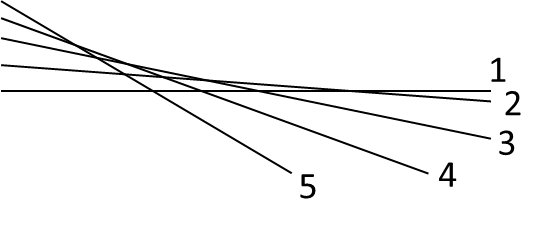

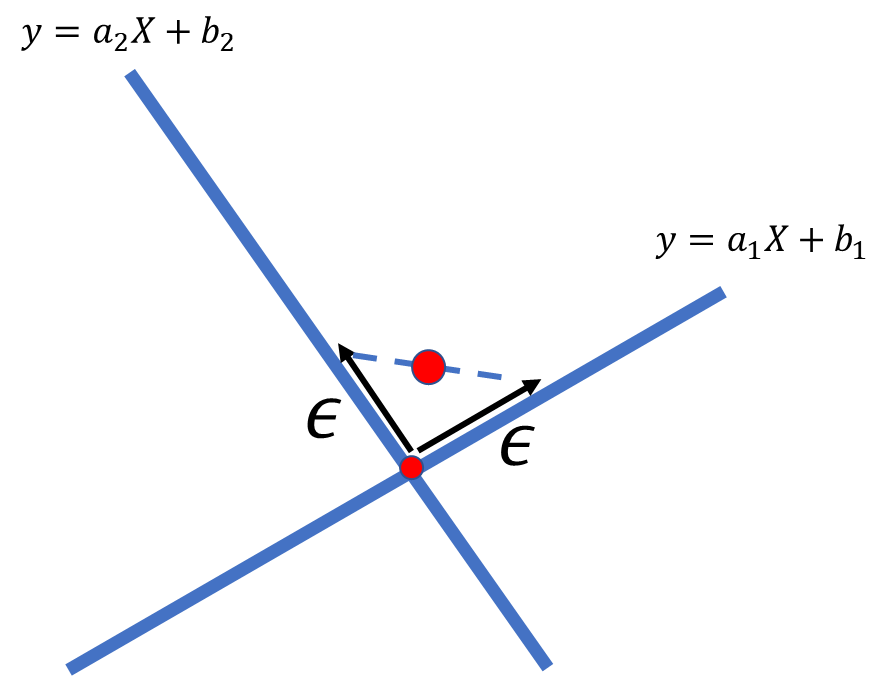


Supplementary Figure 8 **–** **(Left)** **Number of 2d cells created by k lines.** Bottom vertex of each cell is assigned to it in a one to one correspondence. The angle between the cell and the bottom vertex is colored in green. A horizontal line is added to count non-finite cells on the bottom. Total count of cells is $\left( \begin{aligned} k \\ 2 \end{aligned} \right)+\left( \begin{aligned} k \\ 1 \end{aligned} \right)+1$. **(Middle) Constructive process to illustrate tightness of result.** Adding lines iteratively we can carefully place them in such a way as to ensure that their intersection generates exactly $\left( \begin{aligned} k \\ 2 \end{aligned} \right)+\left( \begin{aligned} k \\ 1 \end{aligned} \right)+1$ cells. **(Right) implementation of** $\boldsymbol{smH}\boldsymbol{G}^{\boldsymbol{sample}}$ **illustrated in 2D.** Compute plane intersection of 3 planes. In general position these intersect in a point. For each plane, traverse from the intersection a distance of $\epsilon$ along the gradient of the plane in the direction of $y$ dimension. Average the 3 resulting points to yield a pivot inside the cell.

*Theorem III Proof.* By induction –

Basis – For $k$=1 $\left( \begin{aligned} k \\ 3 \end{aligned} \right)+\left( \begin{aligned} k \\ 2 \end{aligned} \right)+\left( \begin{aligned} k \\ 1 \end{aligned} \right)+1=2$ and indeed, a single plane (half-space) divides the space into two halves.

Inductive step – Suppose that $k$ planes have already been added and that the induction hypothesis holds, adding the $k+1$ plane intersects with the first $k$ planes, forming $k$ “new” lines on the $k+1$^th^ plane. From Theorem 1 these lines divide the $k+1$^th^ plane to $\left( \begin{aligned} k \\ 2 \end{aligned} \right)+\left( \begin{aligned} k \\ 1 \end{aligned} \right)+1$ 2D cells. Consequently, each such 2D cell splits a 3D cell in two and adds this amount to the total cell count. Let $R_{k+1}$ be the recurrence relation defining the maximum number of cells formed by $k$ planes,

$$R_{\left( k+1 \right)}\leq R_{\left( k \right)}+\left( \begin{aligned} k \\ 2 \end{aligned} \right)+\left( \begin{aligned} k \\ 1 \end{aligned} \right)+1\leq\left( \begin{aligned} k \\ 3 \end{aligned} \right)+\left( \begin{aligned} k \\ 2 \end{aligned} \right)+\left( \begin{aligned} k \\ 1 \end{aligned} \right)+1+\left( \begin{aligned} k \\ 2 \end{aligned} \right)+\left( \begin{aligned} k \\ 1 \end{aligned} \right)+1=\left( \left( \begin{aligned} k \\ 3 \end{aligned} \right)+\left( \begin{aligned} k \\ 2 \end{aligned} \right) \right)+\left( \left( \begin{aligned} k \\ 2 \end{aligned} \right)+\left( \begin{aligned} k \\ 1 \end{aligned} \right) \right)+\left( \left( \begin{aligned} k \\ 1 \end{aligned} \right)+1 \right)+1=\left( \begin{aligned} k+1 \\ 3 \end{aligned} \right)+\left( \begin{aligned} k+1 \\ 2 \end{aligned} \right)+\left( \begin{aligned} k+1 \\ 1 \end{aligned} \right)+1$$

QED.$∎$


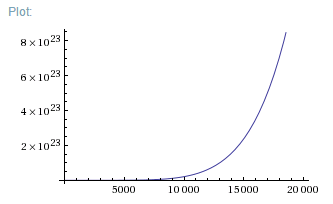


Supplementary Figure 9 **–** **Number of potential different order inducing cells in 3D as a function of number of points.** Showing the intractability of the polynomial problem. X axis denotes the number of points (Genes), and Y-axis denotes the maximum number of cells formed by the bisecting planes between them i.e. $\left( \begin{aligned} \left( \begin{aligned} n \\ 2 \end{aligned} \right) \\ 3 \end{aligned} \right)+\left( \begin{aligned} \left( \begin{aligned} n \\ 2 \end{aligned} \right) \\ 2 \end{aligned} \right)+\left( \begin{aligned} \left( \begin{aligned} n \\ 2 \end{aligned} \right) \\ 1 \end{aligned} \right)+1$.

1. ***C. crescentus* NMDS quality controls:** in Supplementary Figure 10 we provide further detail on the quality controls performed during NMDS linear embedding on a sample dataset.


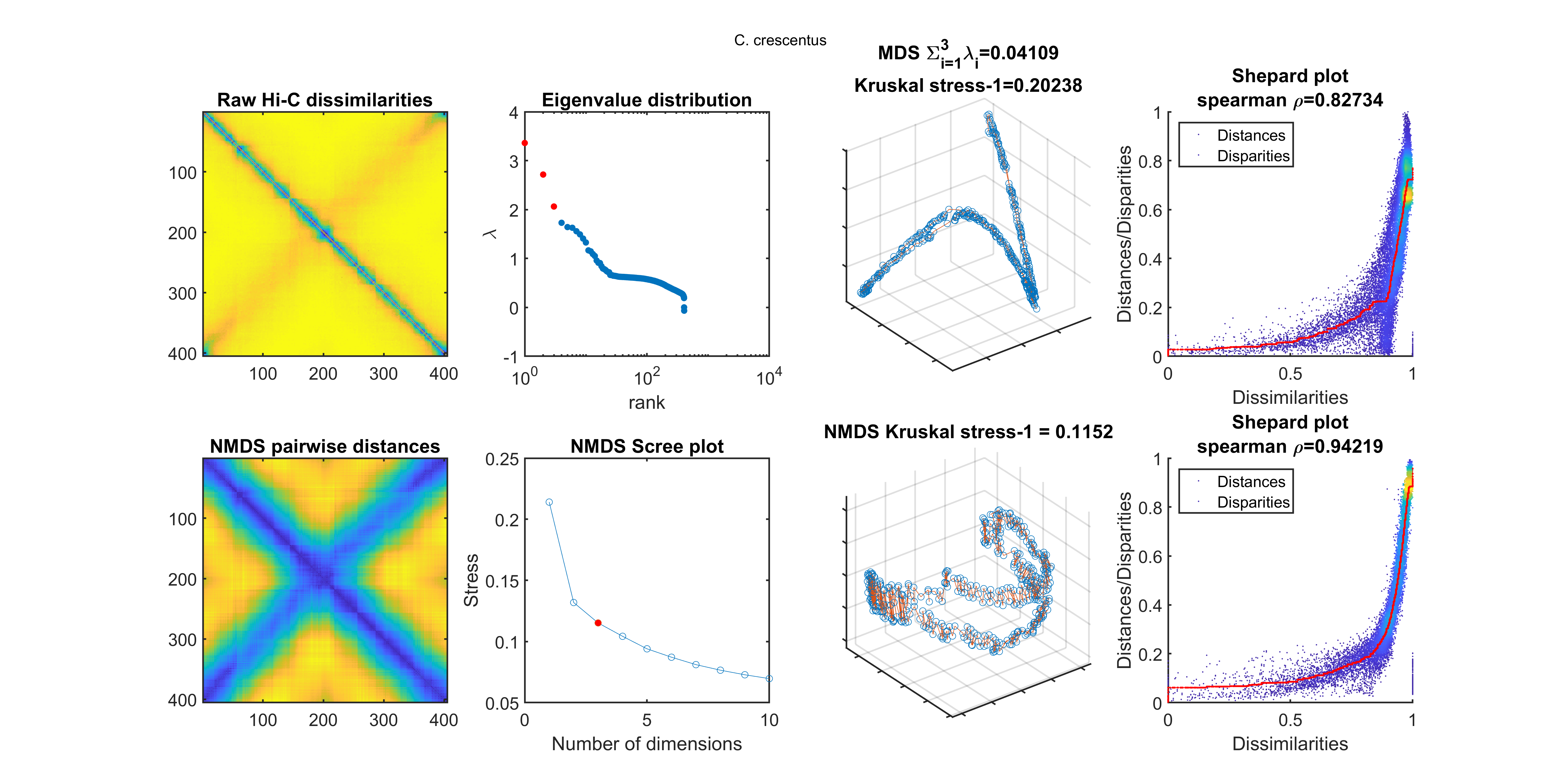


Figure 10 – Subplots are numbered top-to-bottom, left-to-right. (Top) MDS dimensionality reduction steps: 1) Raw Hi-C input matrix is transformed to represent a dissimilarity matrix. 2) Hi-C matrix is projected to a Euclidean space with the centralized Gram matrix. Eigenvalues of the resulting matrix are shown ranked by magnitude. Top 3 eigenvalues, corresponding to a 3D linear projection into a Euclidean space of Hi-C data, are colored in red and show a ‘knee’, hinting at an intrinsic manifold dimensionality in this dataset. 3) MDS 3D embedding result represented by the first 3 eigenvectors with corresponding largest eigenvalues. Normalized sum of eigenvalues (measure of captured variance from linear projection) and Kruskal stress-1 criterion (measure of violations of monotonicity between distances and dissimilarities) values are displayed in title. 4) Shepard plot showing correlation between dissimilarities in the Hi-C data, and distances in the resulting MDS 3D embedding. Disparity line indicates deviations from monotonicity, in resulting embedding. Point cloud is overlaid with a density plot. Spearman rank correlation between dissimilarities and distances, ρ, is displayed in title. (Bottom) NMDS dimensionality reduction steps initialized from the MDS solution: 5) pairwise distances in the resulting NMDS embedding (later visualized in subplot 7), a distinct ‘cross’ pattern emerges that was less visible in the raw Hi-C dissimilarities. 6) A Scree plot showing the impact of selected target dimensionality on the resulting Kruskal stress values shows a ‘knee’ at 3 dimensions. 7) the resulting NMDS embedding and corresponding Kruskal stress. 8) Shepard plot showing correlation between dissimilarities in the Hi-C data, and distances in the resulting 3D NMDS embedding. We see a clear improvement on the spearman ρ compared to the MDS embedding.

1. **A comparison between bisector and Voronoi tessellations:**


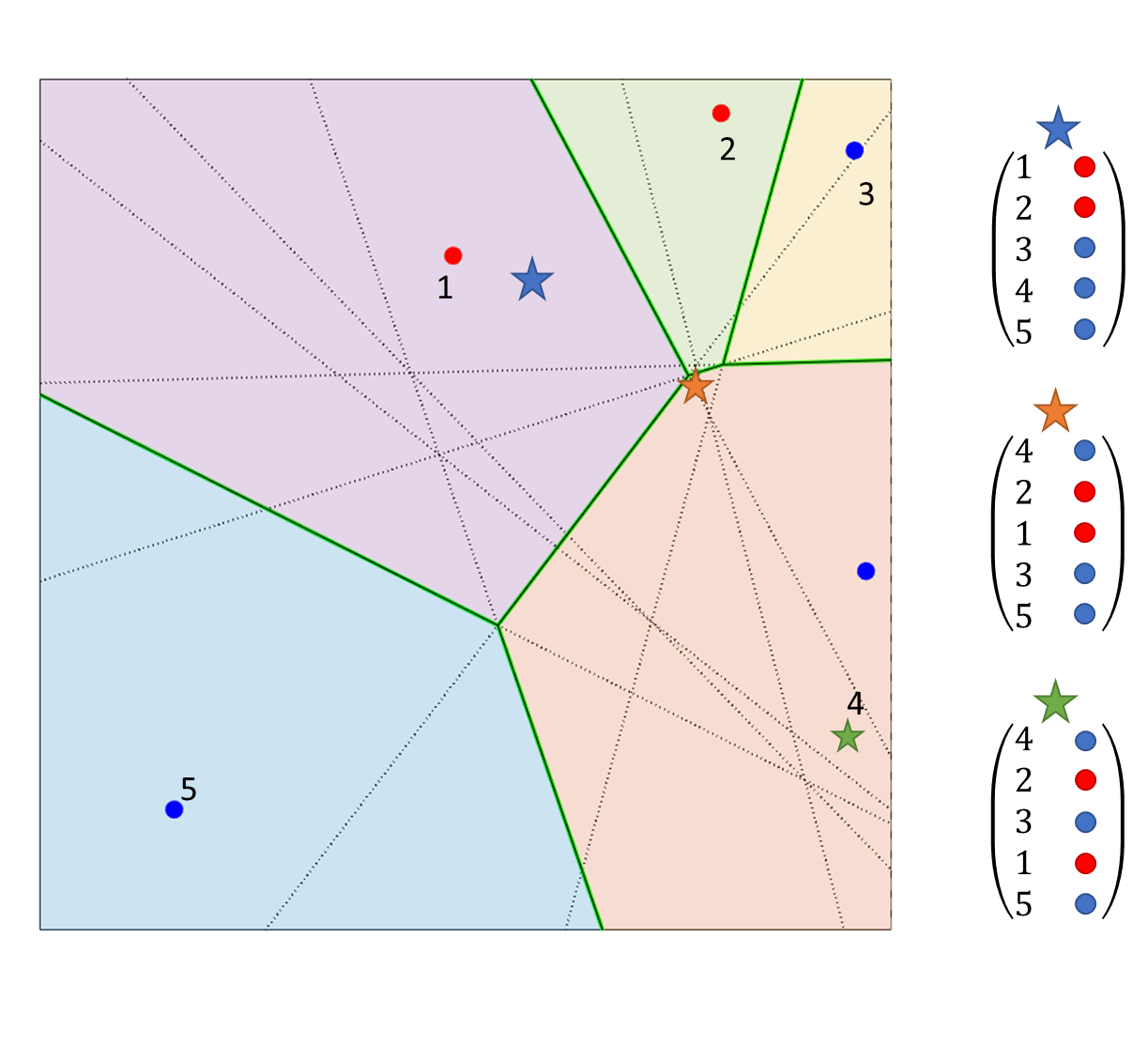


Figure 11 an illustration of bisector tessellation from five points in 2D. Bisectors are presented as dashed black lines. A Voronoi tessellation is induced by an intersection of a subset of bisectors, highlighted in green. Voronoi cells, represented as differently colored polygons, are cells that induce different rankings on the original points such that neighboring cells have a different point at the top of the ranked vector. An example of three pivots inducing different rankings are shown as blue, green and yellow stars. The induced ranked point ids and corresponding labels are shown on the right. Running $smHG$ at the granularity of Voronoi cells would require deciding on a specific pivot for each cell. This example shows that selection of different pivots within a Voronoi cell can have dramatic impact on the ranking and corresponding mHG enrichment results.

1. **Pairwise empirical comparisons among methods:** This figure provides a breakdown of the main results (Supplementary 8) comparing these across evaluated methods. This result empirically confirms that the bead approach is insufficient for detecting some co-localization events that are detected by the grid method.


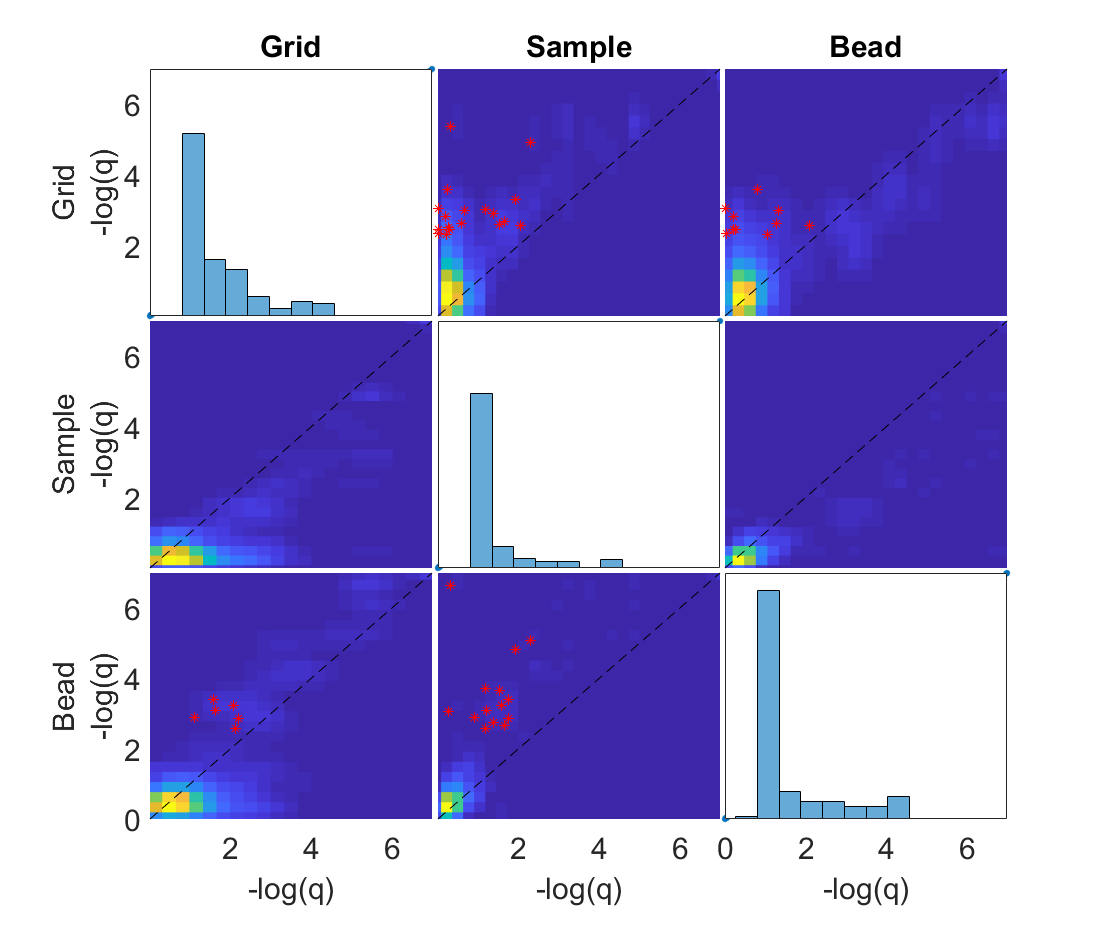


Figure 12 A density plot of every experiment’s resulting Q values in a pair of methods (indicated by the row and column labels). Red asterisks represent a single evaluation where the method labeled by the row detected (Q<0.1) a potential discovery that the method indicated by column did not. Importantly, we observe potential discoveries that would remain undetected by the bead-based method and vice-versa, showcasing that these methods complement one another.

1. **Comparison between smHG and permutation test on raw Hi-C read counts.**


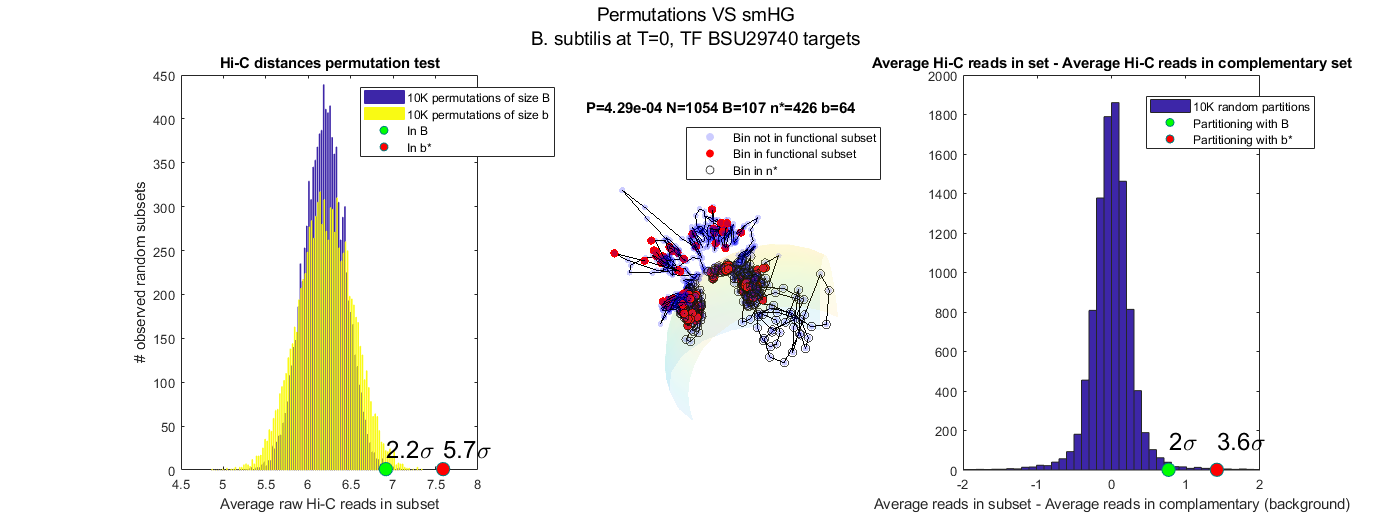


Figure 13 Comparing permutation test with smHG result in B. subtilis (t=0 in timecourse) for a functional group of TF targets (BSU29740): Left) distributions of mean pairwise distances between groups of different sizes are shown in blue and yellow histograms. Correspondingly, the mean pairwise distances between bins in the aforementioned functional group (of size B) is in green. While these are relatively co-localized and are within the 999^th^-quantile, smHG was able to uncover a substantially more co-localized subset (of size b). While both results appear significant, the green result would not be reported when correcting for hundreds of multiple hypotheses. Middle) a 3D embedding of the Hi-C dataset, bins in B are labled in red, bins in b are the ones that also have a dark circle around them, and are within the translucent sphere to the right. We see that this subset is substantially more clustered. Right) We plot a distribution of 10K random partitions of the genome into two, complementary sets, and compute the difference between the average number of reads in both sets.
